# Surface and Bulk Chemistry of Mechanochemically Synthesized Tohdite Nanoparticles

**DOI:** 10.1021/jacs.2c02181

**Published:** 2022-05-23

**Authors:** Jacopo De Bellis, Cristina Ochoa-Hernández, Christophe Farès, Hilke Petersen, Jan Ternieden, Claudia Weidenthaler, Amol P. Amrute, Ferdi Schüth

**Affiliations:** Max-Planck-Institut für Kohlenforschung, Kaiser-Wilhelm-Platz 1, D-45470 Mülheim an der Ruhr, Germany

## Abstract

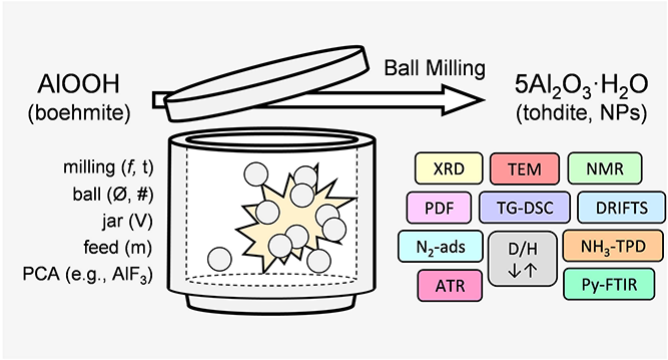

Aluminum oxides, oxyhydroxides, and hydroxides are important in different fields of application due to their many attractive properties. However, among these materials, tohdite (5Al_2_O_3_·H_2_O) is probably the least known because of the harsh conditions required for its synthesis. Herein, we report a straightforward methodology to synthesize tohdite nanopowders (particle diameter ∼13 nm, specific surface area ∼102 m^2^ g^–1^) via the mechanochemically induced dehydration of boehmite (γ-AlOOH). High tohdite content (about 80%) is achieved upon mild ball milling (400 rpm for 48 h in a planetary ball mill) without process control agents. The addition of AlF_3_ can promote the crystallization of tohdite by preventing the formation of the most stable α-Al_2_O_3_, resulting in the formation of almost phase-pure tohdite. The availability of easily accessible tohdite samples allowed comprehensive characterization by powder X-ray diffraction, total scattering analysis, solid-state NMR (^1^H and ^27^Al), N_2_-sorption, electron microscopy, and simultaneous thermal analysis (TG-DSC). Thermal stability evaluation of the samples combined with structural characterization evidenced a low-temperature transformation sequence: 5Al_2_O_3_·H_2_O → κ-Al_2_O_3_ → α-Al_2_O_3_. Surface characterization via DRIFTS, ATR-FTIR, D/H exchange experiments, pyridine-FTIR, and NH_3_-TPD provided further insights into the material properties.

## 1 Introduction

Oxides and hydroxides of aluminum play a central role in various fields of application, as they exist in many different forms with as many different properties.^1^ For instance, apart from their use as a raw material for aluminum metal production, aluminum hydroxides are utilized in the manufacture of paper, paint, glass, ceramic glazes, pharmaceuticals, toothpaste, and fire retardants.^2^ Moreover, aluminum hydroxides are the most direct precursors for other aluminum compounds, including various aluminum oxide forms, via thermal decomposition.^3^ Aluminum oxides also have numerous applications as refractories, advanced ceramics, composites, polishing and grinding tools, catalysts, and catalyst supports.^2^ As a result, the research on aluminum hydroxides and oxides has been and still is a very active area of inorganic chemistry and materials science.

Aluminum oxides and hydroxides have rich chemistry due to their various and different forms. Briefly, aluminum hydroxides can be divided into the trihydroxides (e.g., gibbsite, bayerite, and nordstrandite) and oxide hydroxides (e.g., boehmite, diaspore, and tohdite).^4^ Several crystallographic forms of aluminum oxides are known, but corundum (α-Al_2_O_3_) is thermodynamically the most stable. The other forms (e.g., γ, δ, η, θ, χ, κ, ρ), frequently referred to as “transition” or “activated” aluminas, arise from the thermal decomposition of aluminum trihydroxides and oxide hydroxides under varying conditions.^5^ Subtle structural differences account for the relatively similar properties of aluminum hydroxides and oxides as technical materials, including hardness, resistance to chemicals, thermal properties, electrical conductivity, and optical properties.

Notably, even minor differences, especially for activated aluminas, are sufficient to cause the wide-ranging diversity of some surface properties (e.g., acidity/basicity, the density of active sites, degree of hydroxylation, thermal and hydrothermal stability), which are most relevant in catalysis and adsorption technologies.^6,7^ For instance, it has been reported that metal dispersion, i.e., of Rh and Pd(Ag), can be influenced by the type of alumina phase used as the support.^8,9^ It has also been observed that the catalytic behavior of alumina phases in the methanol-to-dimethylether processes depends on the textural properties, crystallinity, and total amount of acid sites of the alumina phase used as the catalyst.^10−12^ Among others, γ-Al_2_O_3_ is the best candidate for methanol dehydration processes; however, its use is limited mainly by the poor hydrothermal stability.^13,14^ α-Al_2_O_3_ would be a more suitable choice, being more robust toward chemical weathering than γ-Al_2_O_3_, but only if the requirements of surface acidity and high surface area are met.^15^

Most of these materials are relatively well studied and known, but much less information is available for tohdite. The main reason for this lies in the harsh conditions required for its synthesis. Otherwise, tohdite is found in nature only as a minor component of some bauxite ores.^16^

If tohdite as an exotic aluminum oxide hydroxide should be studied or used, it is formed by the hydrothermal treatment of suitable alumina precursors (e.g., η-Al_2_O_3_) under rather harsh conditions.^17^ Temperatures around 460 °C and pressures up to 300 atm are generally required. Furthermore, the use of mineralizers (e.g., AlF_3_) is mandatory to prevent the formation of other stable phases in the “alumina–water” phase diagram (e.g., α-Al_2_O_3_). Spectroscopic evidence and thermogravimetry confirmed the existence of what the authors referred to as “crystalline water” in tohdite, compatible with the formula 5Al_2_O_3_·H_2_O, which is the one typically used to refer to tohdite since it was first synthesized.^18^ On the other hand, the structure of tohdite is best described by the formula Al_10_O_14_(OH)_2_ (vide infra), which also most faithfully reproduces its oxide hydroxide nature.^19^ The structure of tohdite was originally recognized as similar to that of akdalaite, a naturally occurring mineral characterized by a slightly different composition (i.e., 4Al_2_O_3_·H_2_O plus minor amounts of Fe_2_O_3_, BeO, ZnO, and MgO).^20^ Consequently, the terms tohdite and akdalaite have often been improperly interchanged in the literature, leading to some confusion. Nevertheless, the most reliable structural model for tohdite is from Parise et al.,^19^ as a result of thorough characterization of tohdite samples with methods such as single-crystal X-ray diffraction (XRD), neutron powder diffraction, and solid-state NMR. The model essentially reproduces the one reported by Yamaguchi et al.^21,22^ but also describes the environment surrounding the H atoms.

Interestingly, Amrute et al. reported tohdite as a possible intermediate in the mechanochemically induced dehydration of boehmite to high-surface-area corundum.^23^ This suggested that suitable conditions could be found to synthesize tohdite with high selectivity by a relatively simple procedure. Results of the optimization of the mechanochemical synthesis are reported herein, resulting eventually in the synthesis of almost phase-pure tohdite nanopowders, which were thoroughly characterized. The following results and discussion part are organized as follows. In Section 2.1, the literature background on mechanochemically induced transformations in the alumina–water phase diagram is documented. In Section 2.2, the dehydration of boehmite to corundum upon ball milling is described, including possible intermediates and byproducts. Optimization of reaction conditions resulted in the synthesis of powders with a high content of tohdite, which were characterized in-depth, as discussed in Section 2.3. In Section 2.4, the effect of AlF_3_ as a process control agent is described. Finally, Sections 2.5 and 2.6 deal with the thermal stability of the samples and surface characterization results, respectively.

## 2 Results and Discussion

### 2.1 Background Information on Mechanochemically Induced Transformations in the Alumina–Water Phase Diagram

There is some evidence to suggest that mechanochemically induced transformations in the so-called alumina–water phase diagram follow the well-known thermal pathways. For instance, Tonejc et al. observed that the dehydration of γ-AlOOH (boehmite) to α-Al_2_O_3_ (corundum) upon ball milling proceeds through the intermediate formation of transition alumina phases according to the well-known temperature-induced transition sequence, including γ- (or δ-) Al_2_O_3_.^24^ Similar considerations were extended to the case of γ-Al(OH)_3_ (gibbsite) when subjected to the mechanochemical treatment as the transformation to corundum was anticipated by the formation of transition aluminas, supposedly χ- and κ-Al_2_O_3_ one after the other.^25^ Independently, Kostić et al. observed that the transformation of γ-Al_2_O_3_ to α-Al_2_O_3_ could proceed through the sequential transition to δ-Al_2_O_3_ and then θ-Al_2_O_3_ before α-Al_2_O_3_ upon ball milling.^26^ However, these accounts must be approached with some caution because the structural similarity of transition aluminas and their poor crystallinity make it difficult to distinguish alumina phases only from the XRD patterns of milled powders (as often happens).^5^ Contradictory results are not even unprecedented in the literature.^27^ Besides, as the outcome of a mechanochemical synthesis can be affected by many technical parameters, transformation sequences may sometimes go unnoticed (or not observed at all) because experimental conditions are simply unsuitable.^28−31^ Finally, the occasional formation of exotic aluminum oxide hydroxide phases (e.g., tohdite and diaspore) introduces an additional level of complication in the mechanistic understanding of transformations induced by ball milling. In a recent study, which sets out to determine conditions suitable for the synthesis of high-surface-area α-Al_2_O_3_ from the dehydration of boehmite, Amrute et al. reported on low levels of diaspore and tohdite in the milled powders.^23^ Notably, tohdite was recognized as a persistent component of milled powders, and only extensive milling could virtually reduce its presence to zero (or long calcination at elevated temperatures). This evidence was found as consistent with the relative thermodynamic stability of tohdite and corundum, considering increased surface energy of nanoparticles and surface stabilization by hydroxylation.

### 2.2 Evolution of Tohdite from Boehmite upon Ball Milling

During the investigation of the effect of multiple reaction parameters on the mechanochemical synthesis of high-surface-area α-Al_2_O_3_ from boehmite in a planetary ball mill, specific conditions were found under which the formation of appreciable fractions of tohdite could be easily monitored. These conditions imply mild ball milling at frequencies as low as 500 or 400 rpm. As a side remark, the results obtained when other parameters were screened (e.g., milling frequency, number and size of milling balls, the material of milling equipment, jar capacity, ball-to-powder ratios) are not reported herein for brevity, since in such experiments tohdite contents were substantially lower. Instead, only the results achieved under conditions where tohdite formation could be readily monitored are discussed.

**Figure 1:**
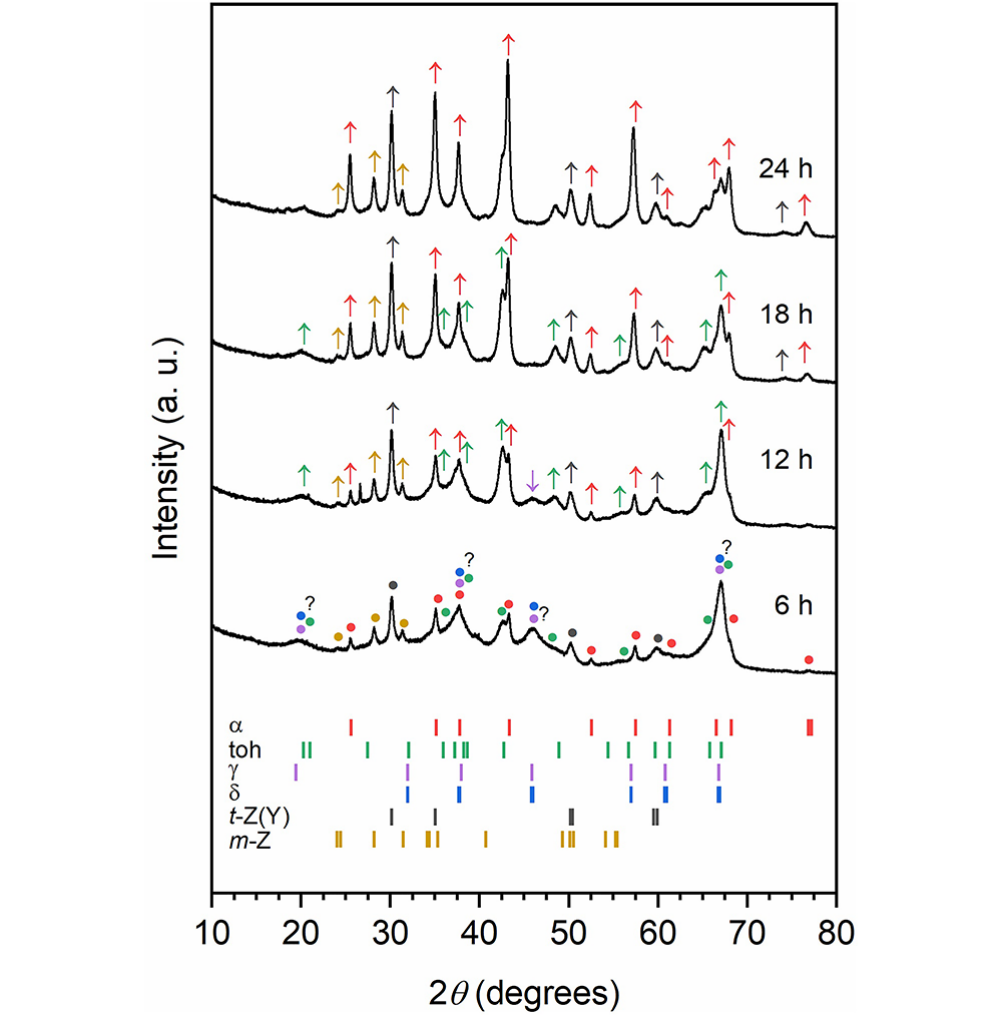
XRD patterns of the materials resulting from the dehydration of γ-AlOOH (boehmite) after 6, 12, 18, and 24 h of milling at 500 rpm, as specified. The most prominent reflections of the crystalline phases present in the sample are marked with symbols (solid circles) according to the following color code: α-Al_2_O_3_ (corundum, red), 5Al_2_O_3_·H_2_O (tohdite, green), γ-Al_2_O_3_ (purple), δ-Al_2_O_3_ (blue), *t*-ZrO_2_ (stabilized, gray), and *m*-ZrO_2_ (yellow). The arrows pointing up or down indicate an increase or decrease in the intensity of the diffraction lines (same color code as before). The position of the most intense diffraction lines (>10%) for the same set of phases is reported in the lower box (ICDD PDF-2 database).

In detail, when milling was carried out at a frequency of 500 rpm (see Section 4 for more information), the transformation of boehmite to corundum stretched over about 24 h. Boehmite was not detected anymore by XRD already after 6 h of milling; instead, some form of transition alumina as the dominant crystalline phase was present (Figure 1). A careful study of the positions and relative intensities of detected reflections suggests γ- or δ-Al_2_O_3_ as the most probable phases. Tohdite and corundum are also found in the solid mixture, as many characteristic reflections match. Upon further milling, i.e., up to 18 h, the reflections typical for tohdite become more evident, while those for γ- (or δ-) Al_2_O_3_ fade into the background. On the other hand, the amount of corundum grows steadily during ball milling, and after 24 h, corundum becomes the dominant crystalline phase in the sample. At any time, zirconia was also present in the milled powders due to abrasion of the milling jar and media, i.e., up to 6.5 wt % after 24 h of milling according to the energy-dispersive X-ray spectroscopy (EDX) bulk elemental analysis.

**Figure 2:**
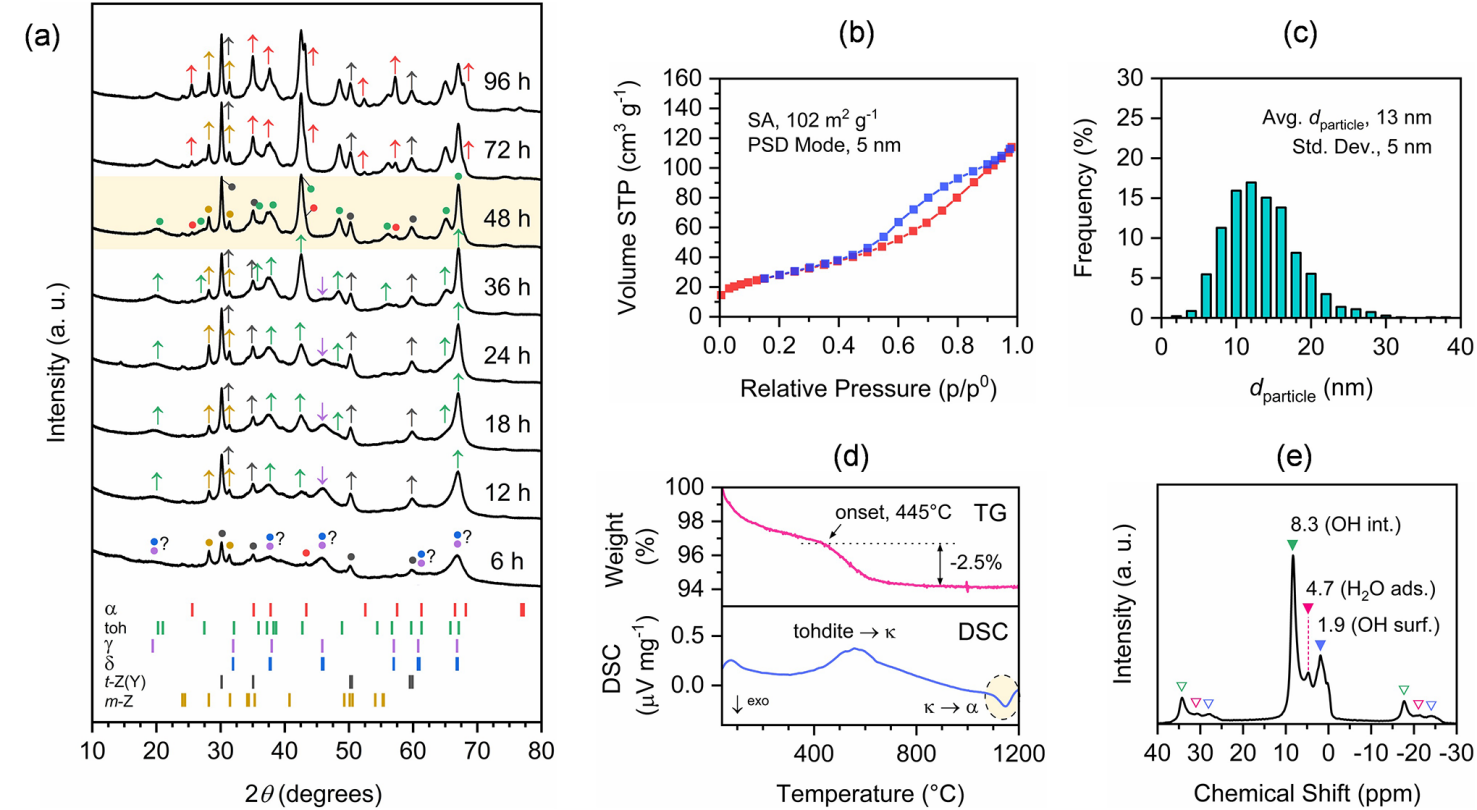
(a) XRD patterns of the materials resulting from the dehydration of boehmite (γ-AlOOH) upon 6, 12, 18, 24, 36, 48, 72, and 96 h of milling at 400 rpm, as specified. The most prominent reflections of the crystalline phases present in the sample are marked with symbols (solid circles) according to the following color code: α-Al_2_O_3_ (corundum, red), 5Al_2_O_3_·H_2_O (tohdite, green), γ-Al_2_O_3_ (purple), δ-Al_2_O_3_ (blue), *t*-ZrO_2_ (stabilized, gray), and *m*-ZrO_2_ (yellow). The arrows pointing up or down indicate an increase or decrease in the intensity of the diffraction lines in the time profile (same color code as before). The position of the most intense diffraction lines (>10%) for the same set of phases is reported in the lower box (ICDD PDF-2 database). (b–e) Characterization data for the material resulting from 48 h of milling. (b) N_2_-adsorption (red) and desorption (blue) isotherms. Specific surface area (SA, BET method) and most probable (mode) pore size in the distribution (PSD, BJH method) are reported. (c) Particle size distribution (histogram) with the average particle size (diameter) and standard deviation. (d) TG and DSC curves measured after activation at 350 °C for 1 h under a high vacuum. The weight loss observed from the onset of the transformation of tohdite to κ-Al_2_O_3_ is highlighted. (e) ^1^H MAS NMR spectrum with peak assignments. Solid and empty symbols relate central peaks with the corresponding first-order sidebands in the given chemical shift range.

An even clearer time profile for the transformation was obtained at a milling frequency of 400 rpm because lowering the milling frequency slowed down the transformation rate without affecting the reaction pathway (Figure 2a). Transition aluminas (e.g., γ- or δ-Al_2_O_3_) are again the first to form from boehmite after about 6 h of milling. Then, the formation of tohdite is observed after 12 h of milling. Interestingly, upon further milling, tohdite appears to gradually take the place of the transition aluminas, and after 48 h, tohdite represents the dominant crystalline phase of the sample. The alternation from transition aluminas to tohdite can be readily appreciated by the increase in the intensity of the reflection centered around 42.7°, typical for tohdite, and the weakening of the reflection centered around 45.8°, characteristic for both γ- and δ-Al_2_O_3_. This evidence would suggest that tohdite could form from a transition alumina phase in the mechanochemically induced dehydration of boehmite to corundum under suitable conditions. However, corundum is not the last to form. As before, trace amounts of corundum were already detected after 6 h of milling, indicating that corundum could form at any stage of the process. This notion is corroborated by the evidence that most transition aluminas appear to be suitable precursors for corundum in the mechanochemical synthesis, as first reported by Zieliński and co-workers.^31^ On the other hand, the crystallization of corundum only becomes significant when the crystallization of tohdite is complete, i.e., above 48 h of milling, as inferred from the increase in the intensity of corresponding reflections. The higher stability predicted for tohdite under reaction conditions possibly accounts for its early appearance and consolidation during the mechanochemical synthesis.^23^ Nevertheless, the small free-energy difference between tohdite and α-Al_2_O_3_ does not exclude the transformation of tohdite into α-Al_2_O_3_,^23^ particularly under a persistent mechanochemical energy input. Finally, zirconia was again found in all samples but to a lesser extent than before, i.e., up to 4.3 wt % after 24 h of milling and 5.1 wt % after 96 h, due to the milder conditions used.

As a side remark, solid-state ^27^Al NMR spectroscopy was also performed to further study the phase composition of the milled powders, but the study was not conclusive. Spectra were poorly resolved, which is attributed to the high defectiveness and possibly amorphization, not unexpected for milled powders. Together with the simultaneous presence of several phases, these factors translated into an inconclusive analysis.

In summary, these results show that the mechanochemically induced dehydration of boehmite to corundum likely proceeds through the intermediate formation of transition alumina phases, as reported before, and of tohdite. The exact nature of the transition alumina phases appearing during the mechanochemical reaction could not be established; both γ- and δ-Al_2_O_3_ are possible. However, per se, the formation of tohdite suggests that the transformation might not reproduce the well-known thermal routes because no such pathway connecting known aluminum oxide or hydroxide phases and tohdite has ever been reported so far. The relative phase composition of the solid mixture could be easily controlled by tuning experimental conditions. Thus, by lowering the milling frequency, it was possible to reach a state where tohdite was the dominant crystalline phase, with corundum present only in traces, i.e., after 48 h of ball milling at 400 rpm. This strategy is highly appealing because it offers easy access to hard-to-synthesize compounds, including metastable and intermediate phases. Particularly, the preparation of tohdite-rich powders in a fast and completely dry way is a rather exciting result because it is unprecedented and makes tohdite so easily accessible that it could be explored in different applications. The following sections will be dedicated to the in-depth characterization of the material.

### 2.3 Characterization of Tohdite-Rich Nanopowders

The material obtained by milling boehmite for 48 h at 400 rpm (more information in Section 4), which will be referred to as tohdite-rich (nano)powder as follows, was characterized thoroughly. First, the contribution of tohdite to the XRD pattern (Figure 2a) was established by comparing experimental data with a simulated diffraction pattern based on the crystal structure refinement by Yamaguchi et al. (Figure S1a).^22^ Thus, XRD indicated that tohdite is the dominant crystalline phase of the sample, with corundum only present in small amounts. Specifically, tohdite contributes to the XRD pattern with diffraction lines, reasonably centered around the expected 2θ-values with the only exception of the one at approximately 65.1°, visibly shifted to lower 2θ-values (expected, 65.9°). This point will be discussed in detail in the following sections when dealing with the thermal behavior of the sample (Section 2.5). On the other hand, the relative intensity of the reflections does not match the one shown by the simulated pattern. This evidence points to a significant disorder of the measured structure compared to the averaged model on which the simulation is based. It is also important to note that the measured reflections are rather broad, indicating a small size of the coherently scattering crystalline domains. The second most relevant crystalline phase in the sample is zirconia, resulting from the abrasion of the milling equipment. Both tetragonal (stabilized) and monoclinic forms of zirconia were detected via XRD, as represented by very sharp and intense reflections, possibly reproducing a relative composition dependent on the manufacture of milling tools. Despite this, the amount of zirconia in the sample is not elevated. EDX bulk elemental analysis reported 3.2 wt % of Zr in the sample, corresponding to approximately 4.3 wt % of ZrO_2_. Since the milling tools were manufactured using 5.5 wt % of Y_2_O_3_ as a stabilizer, the sample should also include about 0.3 wt % of Y_2_O_3_ (0.2 wt % Y), which was not detected via EDX analysis. As a result, abrasion of the milling equipment accounts for 4.6 wt % of the milled powders. Quantitative analysis from the XRD was not reliably possible, mainly due to the defective nature of the samples and the nanocrystallinity.

Local structural analysis from total scattering X-ray data followed by pair distribution function analysis (PDF) provides more insight into the degree of disordering. Comparison of the experimental PDF with simulated PDFs shows that data evaluation is possible, although complicated by the presence of different phases (Figure S1b). The contribution to the PDF from ZrO_2_ is easy to discriminate from the others. In contrast, tohdite and corundum are expected to exhibit similar PDFs, regardless of the structural differences, as shown by Figure S1b. Still, the experimental PDF fits very well the one simulated for the averaged tohdite structure, at least within near- and mid-range distances (Figure S1c). However, the first strong atom-pair correlation, i.e., around 1.8–2.0 Å (Al–O, first coordination sphere), is much more intense for the experimental PDF than expected. Possibly, a substantial amount of ultra-nano-corundum, that would elude detection via XRD, likely contributes to the phase composition of the sample. Conversely, for more considerable distances, i.e., above 13–15 Å, a significant mismatch is observed, probably due to structural disorder.

Tohdite was also identified via solid-state NMR, particularly ^1^H MAS NMR. As previously reported by Parise et al., the ^1^H MAS NMR spectrum of tohdite should consist of a single narrow central peak at a relatively high chemical shift (i.e., +8.6 ppm from tetramethylsilane).^19^ This signal is attributed to the internal OH groups, possibly involved in H-bonding with neighboring O atoms. For our material, a ^1^H NMR peak was detected in a similar chemical shift range (i.e., +8.3 ppm), as shown in Figure 2e. However, in contrast with the case of Parise et al., where only a small shoulder to the central peak arose, in our case, a more significant contribution was observed from the adsorbed water, regardless of an activation procedure (Figure S2a), and surface hydroxyl groups. Nevertheless, the ratio of tetra- and hexacoordinated Al species, i.e., 0.24 (Table S1), estimated by the careful analysis of the ^27^Al MAS NMR spectrum, appeared to be consistent with the expected value (i.e., 0.25).^32^

Overall, the material is characterized by a relatively high specific surface area of about 102 m^2^ g^–1^, as established via N_2_-sorption experiments (BET method), suggesting that primarily nanoparticles represent the material. This notion was corroborated by the direct observation of the nanoparticles via electron microscopy (Figure S3). Particularly, the material is characterized by nanoparticles of approximately spherical shape (Figure S3c,d) with an average size (diameter) of around 13 nm and a standard deviation of about 5 nm (Figure 2c) randomly close-packed in large aggregates (Figure S3a,b). Particle aggregation possibly accounts for the porosity detected during the N_2_-adsorption/desorption experiments (Figure 2b). Besides, the most probable pore size (i.e., 5 nm according to the BJH method) is compatible with the value expected for the distribution of interparticle voids in a random close packing of spherical particles described by a similar relative standard deviation of sizes.^33^

Upon heating to 1200 °C, the as-synthesized material releases about 10.9 wt % (TG; Figure S4) of water (confirmed by MS) in two steps: first, before 400 °C, possibly due to the release of adsorbed water (^1^H MAS NMR; Figure S2a) and surface reconstruction (e.g., condensation reactions) and then, above 400 °C, in the transformation of tohdite to κ-Al_2_O_3_ (more information in Section 2.5). Since the two events strongly interfere, an exact evaluation of individual contributions to the TG curve profile is impossible for the as-synthesized material. Nevertheless, the profile of the TG curve above 400 °C can be improved upon prior heating at 350 °C for 1 h under a high vacuum (Figure S4). As a result, a more precise evaluation of the amount of water released during the transformation of tohdite to κ-Al_2_O_3_ is possible for the activated material.

The thermogravimetric analysis-differential scanning calorimetry (TG-DSC) experiments carried out on the activated material are also presented in Figure 2d. Notably, above 445 °C, the material experiences a weight loss of about 2.5 wt % due to the transformation of tohdite to κ-Al_2_O_3_ (TG; Figure 2d). Then, above 630 °C, the TG curve slowly establishes a plateau so that about 94.1 wt % of the sample is left at 1200 °C. As a side remark, only α-Al_2_O_3_ and ZrO_2_ are detected via XRD for the sample heated at 1200 °C. Interestingly, whereas the transformation of tohdite to κ-Al_2_O_3_ is endothermic (DSC; Figure 2d), the transition from κ- to α-Al_2_O_3_ (i.e., onset at 1100 °C at a heating rate of 10 °C min^–1^) is characterized by an exothermic signature (Figure 2d, highlighted). The transformation from tohdite to κ-Al_2_O_3_ is observed at lower temperatures than previously reported (i.e., above 700 °C),^34^ possibly due to the nanocrystallinity of the sample. Likewise, the transition from κ- to α-Al_2_O_3_ should be observed at much higher temperatures (i.e., above 1300 °C).^35^ Similar observations for nanocrystalline materials are not unprecedented in the literature.^36,37^

These results are particularly meaningful to evaluate the selectivity of the process toward the formation of tohdite from boehmite as the precursor. Considering the amount of the as-synthesized material left upon heating to 1200 °C (89.1 wt %) and the impurity level (4.6 wt % as ZrO_2_ and Y_2_O_3_), one can estimate an overall content in Al_2_O_3_ of about 84.5 wt % (or 94.8 wt % of the residue at 1200 °C). Accordingly, approximately 89.2 wt % of the activated material should consist of Al_2_O_3_ units. Considering the amount of water released in the transformation of tohdite (activated) to κ-Al_2_O_3_ (i.e., 2.5 wt % from 445 to 630 °C), it is possible to estimate that about 70.7 wt % of activated material (as Al_2_O_3_ units) corresponds to tohdite. This implies that 79.3% selectivity was achieved in the mechanochemically induced dehydration of boehmite toward tohdite formation. However, the previous notion is valid only under the assumption that the formula 5Al_2_O_3_·H_2_O describes well the composition of tohdite. Possibly, the remaining fraction is composed of amorphous material and nanocrystalline corundum.

Interestingly, the tohdite samples proved to be highly stable under hydrothermal conditions. The tohdite phase was found unchanged after 24 h of autoclave treatment at 150 °C in deionized water (H_2_O/Al ratio of about 150). Only the amorphous fraction possibly present in the sample was affected as γ-AlOOH (boehmite) could recrystallize during the hydrothermal treatment (Figure S14). Hence, tohdite was recognized as highly stable toward chemical weathering as α-Al_2_O_3_ (corundum).^15^ This is very appealing for catalysis applications where the exploitation of alumina phases (e.g., γ-Al_2_O_3_) is limited due to their poor stability in water-containing environments.^38^

### 2.4 High-Yield Synthesis of Tohdite Nanoparticles

**Figure 3:**
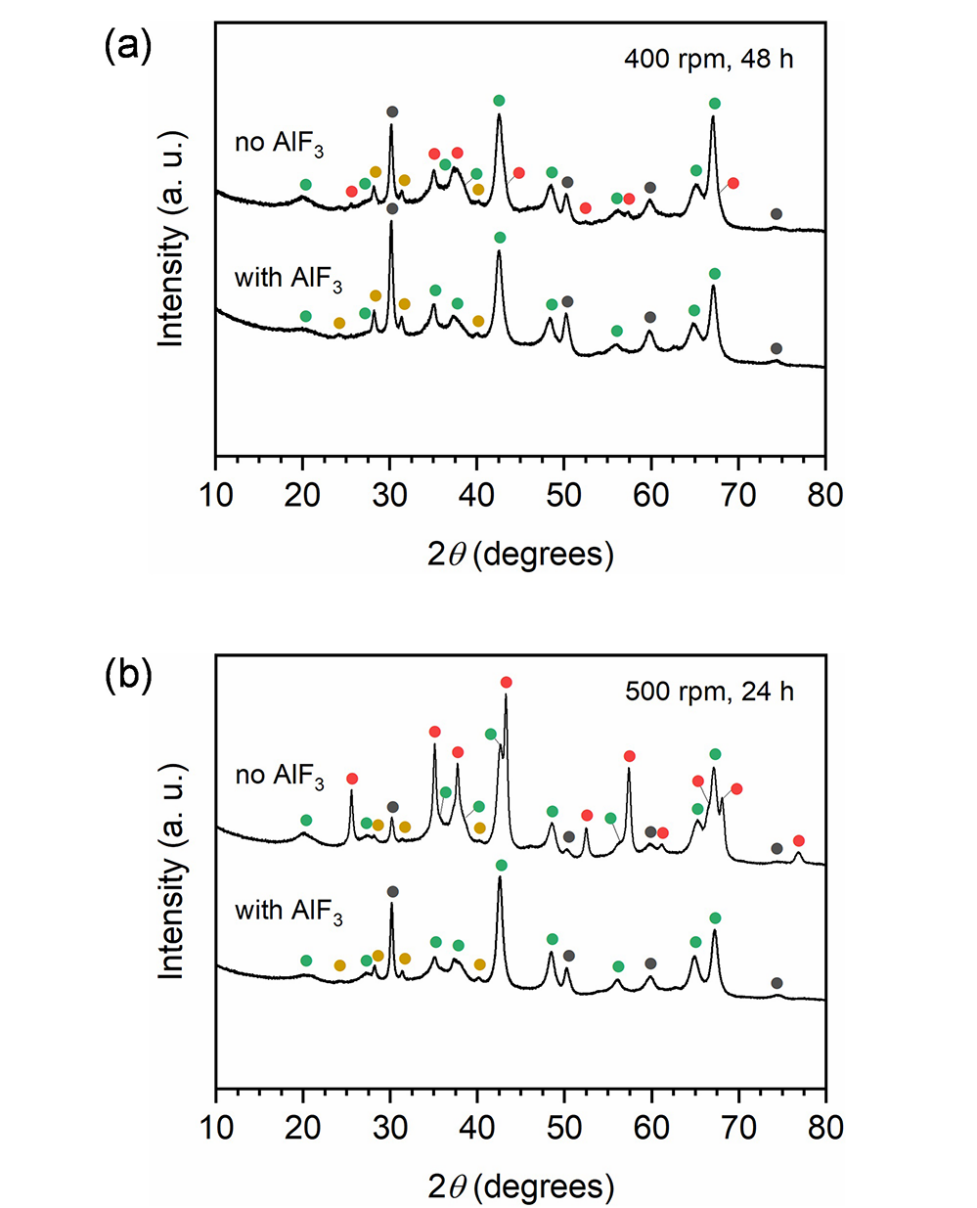
XRD patterns of the materials resulting from 48 h of milling at 400 rpm (a) and 24 h of milling at 500 rpm (b), with or without AlF_3_ in the role of a process control agent. In each case, the most prominent reflections of the crystalline phases present in the sample are marked with symbols (solid circles) according to the following color code: α-Al_2_O_3_ (corundum, red), 5Al_2_O_3_·H_2_O (tohdite, green), *t*-ZrO_2_ (stabilized, gray), and *m*-ZrO_2_ (yellow).

The selectivity of the mechanochemically induced dehydration of boehmite to tohdite is already very high. However, previous studies on tohdite formation following other pathways showed that the selectivity toward tohdite formation over corundum could be greatly improved by introducing a suitable process control agent, such as AlF_3_. The addition of a small amount of AlF_3_ to the milling jar under optimized conditions (i.e., 48 h of milling at 400 rpm) indeed prevented the crystallization of corundum to such an extent that no corundum could be detected anymore in the XRD patterns of milled powders (Figure 3a). An even more revealing result was obtained when the synthesis was repeated under conditions known to favor the formation of corundum (e.g., 24 h of milling at 500 rpm). As clearly shown in Figure 3b, when no process control agent was employed, milling indeed led to the formation of tohdite but also of a large amount of corundum. In contrast, when AlF_3_ was used, no corundum was detected but tohdite only. This is in line with the study of Yamaguchi et al., similarly recognizing AlF_3_ as an effective mineralizer for tohdite formation under hydrothermal conditions so that nucleation of corundum was entirely suppressed when AlF_3_ was used.^17^ Besides, the relative stability of phases followed the order γ-AlOOH (boehmite) <γ-Al_2_O_3_ <5Al_2_O_3_·H_2_O (tohdite) <α-Al_2_O_3_ (corundum), seemingly reproduced upon ball milling.^17^ These results establish unusual parallelism between hydrothermal and mechanochemical synthesis.

It has already been considered that hydrothermal conditions, similar to those in an autoclave, might be encountered during mechanochemical activation (e.g., the mechanochemical treatment of pulps, a collision of two solid samples with a rough surface, or the compression of a liquid in capillary pores).^39^ In particular, it was postulated that the water released during the mechanochemical synthesis of complex oxides from the corresponding hydroxides could contribute to the enhancement of reaction rates for the initiation of hydrothermal processes upon ball milling.^40^ As seen from previous evidence, hydrothermal conditions are possibly generated also in our case.

The role of AlF_3_ as a mineralizer (or process control agent) is crucial to understand the mechanism, which favors either corundum or tohdite formation during the dehydration of boehmite upon ball milling. However, a complete analysis is beyond the scope of the present study, and more advanced characterization methods, including in situ techniques, would be necessary to collect all evidence required for a model. Thus, only the results available in this study will be discussed.

According to the ^19^F MAS NMR spectrum (Figure S5c), several F species coexist in the sample. The chemical shift and the peculiar line profile suggest that F participates in the constitution of several AlF_*x*_(OH)_6–*x*_ octahedral species with predominantly low *x*-values.^41^ This notion is corroborated by the slight increase in hexacoordinated Al species detected via ^27^Al MAS NMR compared to that where no AlF_3_ had been used (Table S1). Nevertheless, the ^27^Al MAS NMR spectra look similar (Figure S5d). Finally, the distribution of F across the material appears homogeneous. At least, EDX line scans of selected nanoparticles (Figure S5b) did not highlight any specific sign of surface segregation.

The presence of F^–^ possibly introduces a certain level of structural distortion, as the position and relative intensity of some of the XRD lines differ from the case where no AlF_3_ had been used (Figure 3a). However, the ^27^Al NMR chemical shifts are surprisingly not affected, unlike the ^1^H NMR chemical shifts. The ^1^H NMR signal for internal OH groups is shifted to lower fields (Table S1), suggesting that F^–^ might interfere with the H-bonding system. Besides, the ^1^H MAS NMR spectrum (Figure S6d) shows a remarkable resemblance with that produced by specific structural motifs of crystalline AlF_*x*_(OH)_3–*x*_ species.^42^

All other features are similar to the material resulting from the synthesis without AlF_3_. In particular, the material is represented by nanoparticles of approximately spherical shape (Figure S5a) with an average size (diameter) of around 14 nm and a standard deviation of about 6 nm (Figure S6b), as before randomly close-packed in large aggregates. The average particle size and standard deviation are consistent with a statistically comparable particle size distribution. Moreover, the sample is characterized by a specific surface area and pore size distribution similar to the material obtained without AlF_3_ as the process control agent (Figure S6a). The TG experiments (Figure S6c) indicated high levels of tohdite in the sample (up to 3.3 wt % loss due to the transformation of tohdite to κ-Al_2_O_3_). However, a precise quantification was complicated by the concomitant release of HF and H_2_O from the decomposition (pyrohydrolysis) of AlF_*x*_(OH)_3–*x*_ species to “AlFO” transition compounds observed in a similar temperature range.^43^ The formation of α-Al_2_O_3_ was pointed out by a very strongly exothermic peak (DSC; Figure S6c) centered around 1085 °C.^44^ Finally, similar was also the behavior under hydrothermal conditions. Particularly, the samples resulting from 24 h of milling at 500 rpm with AlF_3_ as the process control agent were mostly unchanged after 24 h of autoclave treatment at 150 °C (Figure S14). Only a small amount of boehmite formed during the hydrothermal treatment, further corroborating the higher-phase purity of the materials obtained upon milling with AlF_3_. As a side remark, the F content of the samples, as found via the EDX bulk elemental analysis, did not change after the treatment. This evidence supports the notion that F is incorporated in milled powders.

Overall, our study further corroborates the notion that ball milling can serve as a convenient alternative to carry out more sustainable chemical processes. The advantages of mechanochemical methods for the chemical synthesis of organic and inorganic compounds, either as molecular solids or as extended materials, have been extensively reviewed in the past years.^45−48^ In many cases, mechanochemical methods enabled easy access to target chemicals at a lower cost and reduced environmental impact. Mechanochemical syntheses typically proceed directly at room temperature, often without the aid of solvents or specific additives as process control agents, achieving high selectivities for the desired products in a relatively short time. This is especially important when mechanochemistry is applied to the synthesis of inorganic materials as an alternative to customary solution-based techniques, including autoclave syntheses and solid-state high-temperature treatments.^47^ The success in the mechanochemical synthesis of tohdite demonstrates that ball milling can also be used to carry out reactions normally performed with autoclave devices at a lower cost and reduced energy consumption. Besides, ball milling is also a safer option compared to the autoclave experiments. Hence, devices used for the mechanochemical activation of solids may in selected cases become an alternative to the autoclave technology and thus simplify—and additionally at reduced cost—hydrothermal syntheses performed in the industry.^49,50^

### 2.5 Thermal Stability of Tohdite-Rich Nanopowders

The thermal stability of the samples upon calcination was investigated in a wide temperature range, particularly from room temperature up to 1200 °C (TG-DSC). As anticipated, in this range, tohdite first transforms to κ-Al_2_O_3_ (onset at 445 °C according to the TG experiments; Figure 2d). Then, the transition from κ- to α-Al_2_O_3_ is observed (above 1000 °C according to DSC; Figure 2d). XRD and solid-state NMR (^1^H and ^27^Al MAS NMR) were the primary means to investigate the transformation sequence.

**Figure 4:**
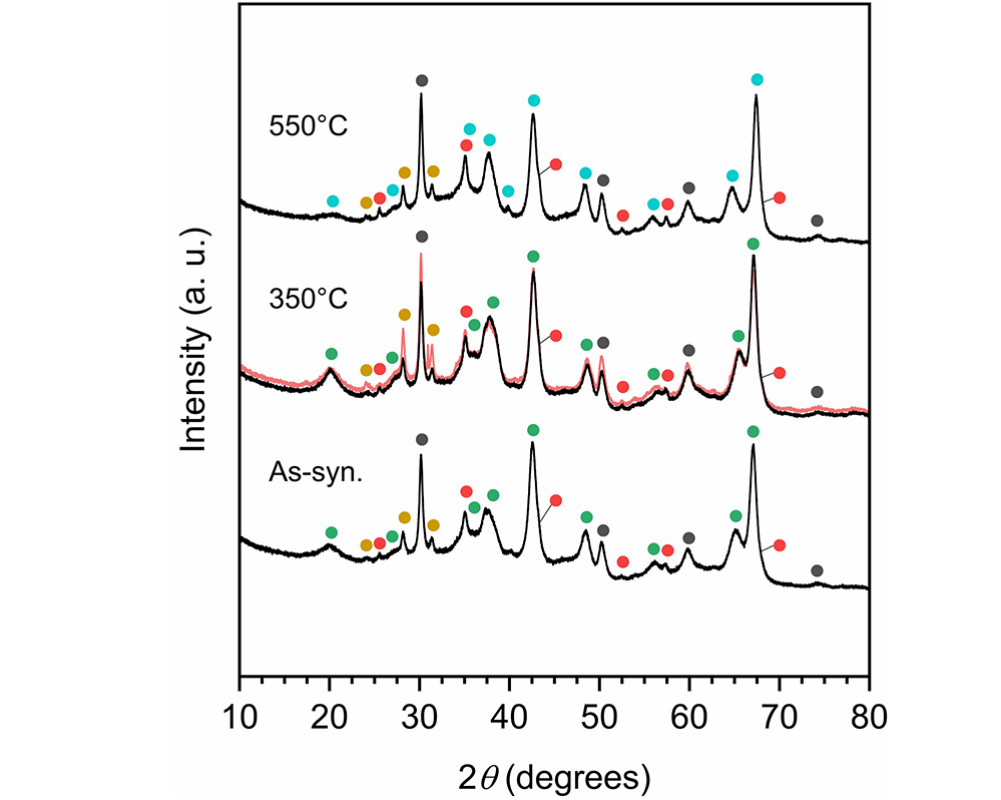
XRD pattern of the as-synthesized material and after calcination at 350 and 550 °C for 10 h, respectively. The red outline represents the XRD pattern of the material resulting from 1 h of annealing at 350 °C under a high vacuum. The most prominent reflections of the crystalline phases present in the sample are marked with symbols (solid circles) according to the following color code: α-Al_2_O_3_ (corundum, red), κ-Al_2_O_3_ (turquoise), 5Al_2_O_3_·H_2_O (tohdite, green), *t*-ZrO_2_ (stabilized, gray), and *m*-ZrO_2_ (yellow).

Independently, it was confirmed that the tohdite phase is stable upon prolonged calcination at 350 °C, i.e., up to 10 h (Figure 4). However, the sample is not entirely idle at the given temperature. In fact, under these conditions, the tohdite phase tends to reconstruct so that the resulting XRD pattern ultimately reveals a better match with simulated data. Mainly, the structural rearrangement is indicated by a shift in the line position and intensity for a selected group of reflections, but most pronouncedly, the reflection at about 65.1° shifts to higher 2θ-values (65.5°). It is probable that defects and structural distortions accumulated during ball milling relax during this curing step. A similar result is obtained when the material is heated at 350 °C under a high vacuum (Figure 4), i.e., under the conditions usually applied to activate the samples before solid-state ^1^H NMR and N_2_-adsorption methods.

The temperature-induced dehydration of tohdite seemingly proceeds without the intermediate formation of κ′-Al_2_O_3_,^51^ but κ-Al_2_O_3_ directly, at least upon calcination at 550 °C for 10 h (Figure 4). Still, it is important to note that the formation of κ′-Al_2_O_3_ was only observed upon heating under a high vacuum and never during conventional heating experiments (calcination).^34^ This transformation was also monitored via ^27^Al solid-state NMR. Although intensification of the contribution from tetracoordinated Al species was observed (Figure S2d–f), the ratio of tetra- and hexacoordinated Al species (i.e., 0.22) is not quite consistent with the expected value for κ-Al_2_O_3_ (i.e., 0.33) (Table S1).^52^ Thus, the study was considered inconclusive. This could be partly due to a not yet fully understood broadening of the line for hexacoordinated Al species, possibly resulting in inaccurate baseline correction.

Calcination at higher temperatures, i.e., from 550 to 800 °C, similarly yielded the same phase, as obvious from the XRD (Figure S7) and ^27^Al solid-state NMR characterization of the samples (Figures S2f and S8b and Table S1). On the other hand, the ^1^H MAS NMR spectra show that signals for OH groups (internal) previously assigned to tohdite, although depleted, are still present (Figures S2c and S8a). This result is somewhat counterintuitive and suggests that some of the tohdite structural motifs could survive prolonged calcination at temperatures as high as 800 °C (more information in Section 2.6).

As a side remark, an interesting feature of the samples obtained from the dehydration of tohdite nanopowders is the relatively high surface area. In fact, the powder is still characterized by a specific surface area of about 88 m^2^ g^–1^ after 10 h at 550 °C and 76 m^2^ g^–1^ after calcination at 800 °C (Figure S9). The specific surface area drops only upon heating above 900 °C (Figure S9) due to the nucleation and growth of α-Al_2_O_3_ (Figure S7). In general, high values of specific surface area and thus small particle size are unusual for high-temperature metastable alumina phases, including θ- and κ-Al_2_O_3_, as it is for the most thermodynamically stable α-Al_2_O_3_. High-surface-area alumina phases, i.e., nanoparticles, often have different properties than the corresponding low-surface-area counterparts. This could be relevant in catalysis, as it has already been demonstrated for α-Al_2_O_3_,^15,53^ and other applications, such as the manufacture of cutting tools and wear resistance ceramics.^54^

### 2.6 Surface and Bulk Characterization of Tohdite Nanoparticles via Infrared Spectroscopy

**Figure 5:**
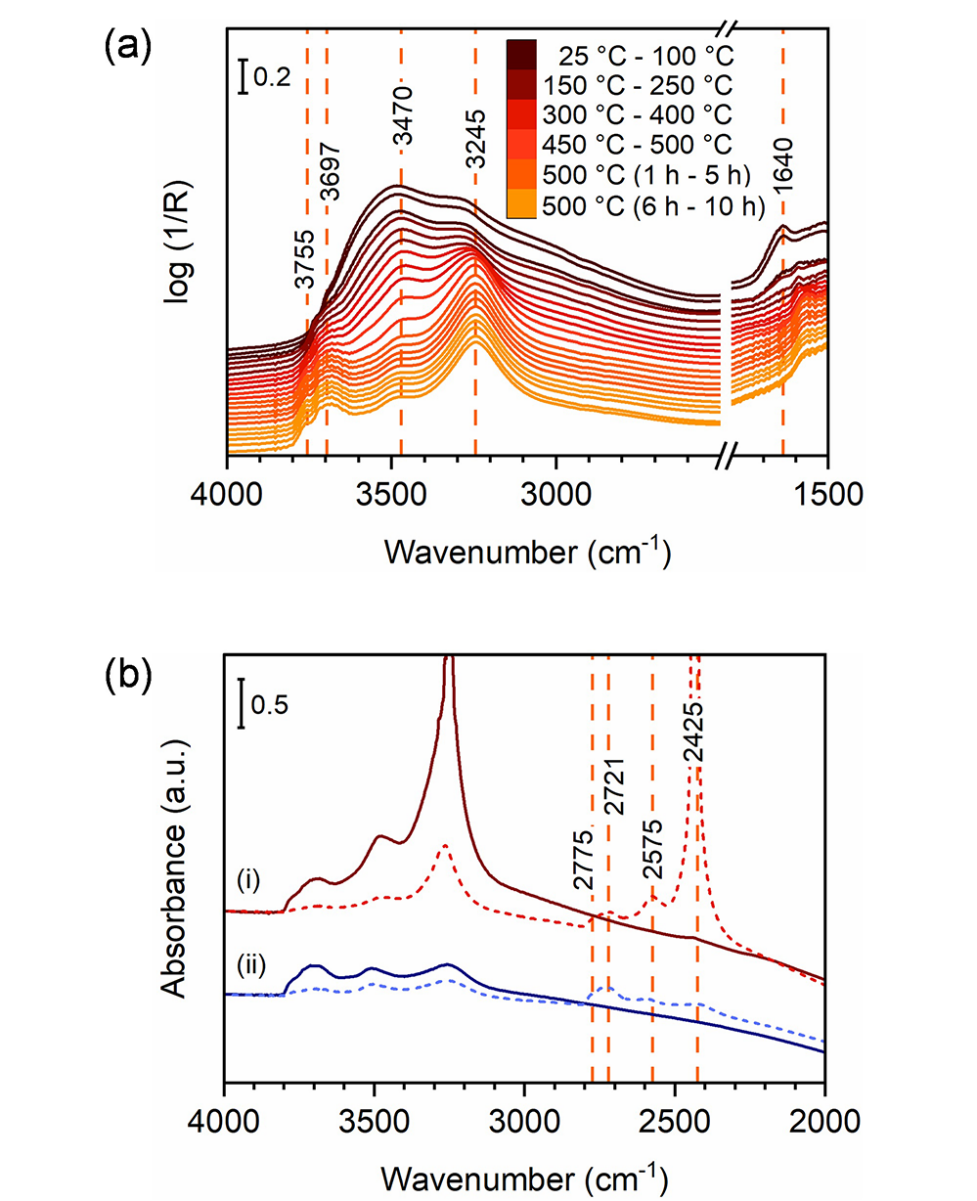
(a) In situ DRIFT spectra of as-synthesized tohdite nanopowders measured at different temperatures, from room temperature to 500 °C. The color guide is given as the inset. The range from 4000 to 2500 cm^–1^ corresponds to the stretching vibration region of the hydroxyl groups. (b) Transmission FTIR spectra of the samples calcined at 350 °C (i) and 550 °C (ii) for 10 h, before (solid line) and after D_2_O treatment (dashed line).

The as-synthesized tohdite-rich sample was thoroughly investigated via diffuse reflectance infrared Fourier transform spectroscopy (DRIFTS). Above all, the stretching of OH groups and their temperature evolution under nitrogen atmosphere were studied in detail (Figure 5a). The presence of physisorbed water interacting with the tohdite hydroxyl groups through H-bonds is evidenced by the characteristic band at ∼1640 cm^–1^ (δ_H2O_). However, upon heating to 200–250 °C, the physisorbed water molecules are removed, and four bands are observed at ∼3755, ∼3697, ∼3470, and ∼3245 cm^–1^. The first two bands, which most pronouncedly intensify with the temperature increase, can be assigned to surface hydroxyl groups. From previous studies on the surface of transitional aluminas, the band at ∼3755 cm^–1^ is most likely related to the terminal hydroxyl groups (type I) bound to Al ions occupying either tetrahedral sites with a cationic vacancy nearby or octahedral sites.^7,55^ On the other hand, the assignment of the band at ∼3697 cm^–1^ is most challenging because it is the subject of some debate across the literature. According to Busca et al., while all bands located above approximately 3700 cm^–1^ are probably due to terminal hydroxyl (type I), the band at ∼3690 cm^–1^ is attributed to the hydroxyl groups bound to two Al atoms (type II).^7,55^ However, this band was also attributed to the OH groups bonded to three Al atoms (type III).^7,56^ In contrast to the previously discussed bands, the one at ∼3470 cm^–1^ is strongly affected by the temperature variation. The intensity of such a band steadily decreases while increasing the temperature to about 400 °C. The presence of some perturbed and temperature-sensitive OH groups, i.e., involved in H-bonding, probably accounts for this behavior. However, since this band does not vanish even after 10 h at 500 °C, contributions from other species cannot be discarded.

Among all existing bands, the most intense signal is at 3245 cm^–1^. Such low frequencies might indicate a substantial perturbation by H-bonds. Nonetheless, the species involved seem to be thermally stable up to ca. 450 °C. Only above 450 °C, the intensity of this band starts to decrease, probably due to the alteration and structural rearrangement of the material to a more compact aluminum oxide structure. In other words, the intensity decrease could point to the transformation of this phase into κ-Al_2_O_3_, which should imply a dehydroxylation process. Besides, the reported temperature would be consistent with the onset for the transformation estimated via the TG-DSC experiments (e.g., 445 °C; Section 2.3). Interestingly, Figure S10 reveals that the four bands survive the transformation from tohdite to κ-Al_2_O_3_. However, their intensity changes considerably and, thus, the ratios between different OH species, with the contribution from the surface OH groups being most predominant after prolonged calcination at 800 °C. The ^1^H MAS NMR spectra showed a similar trend (Figures S2a–c and S8a), as discussed above (Section 2.5). The skeletal region of the FTIR spectrum (Figure S11) also highlights some differences between the samples calcined at 350 and 550 °C, respectively. All of the bands above 680 cm^–1^ have essentially vanished at high temperatures, supporting the notion that alterations of the bulk structure are induced by thermal treatment. Finally, the broadness of the bands could be an indication of a certain level of disorder within the structure, as has already been reported for other transitional aluminas.^7,55^

To get more insights into the nature of the hydroxyl groups of the samples calcined at 350 and 550 °C and possibly discriminate between surface and structural OH species, D/H exchange was performed by exposing the samples to D_2_O vapor at room temperature (more information in Section 4). After deuteration, the OH stretching absorption bands are red-shifted to the OD vibration region (2800–2400 cm^–1^) so that four signals arise at ∼2775, ∼2721, ∼2575, and ∼2425 cm^–1^ (Figure 5b). Accordingly, the overall absorption in the OH region is significantly decreased. Nonetheless, in the case of the sample calcined at 350 °C, even after four cycles, an intense band at ∼3245 cm^–1^ and one less pronounced at ∼3470 cm^–1^ are still observed. Similar observations apply to the sample calcined at 550 °C. This evidence points to the difficulty of isotopically exchanging these species, further suggesting that they could be located in subsurface layers of the material or in the bulk. Similar conclusions were drawn after performing analogous studies on transitional aluminas.^7,57^ On the other hand, the bands at ∼3755 and ∼3697 cm^–1^ almost completely disappear, giving rise to the bands at ∼2775 and ∼2721 cm^–1^. This emphasizes their easy accessibility and corroborates their surface nature.

**Figure 6:**
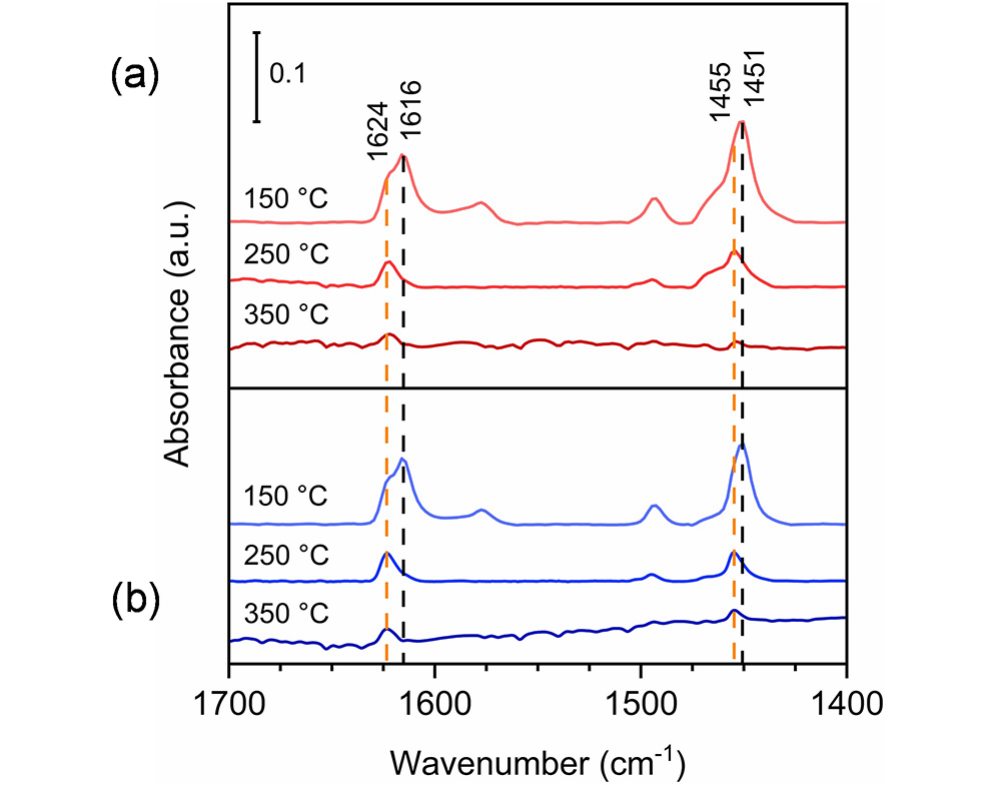
Transmission FTIR spectra of pyridine (ν_CCN_) adsorbed on the samples calcined at 350 °C (a) and 550 °C (b) for 10 h, measured at the given desorption temperatures.

For the samples calcined at 350 and 550 °C, the nature and strength of the possible acid sites were assessed via pyridine adsorption experiments. As clearly illustrated in Figure S12, the presence of a strong basic probe molecule perturbates the bands corresponding to the terminal hydroxyl groups, i.e., at ∼3755 and ∼3697 cm^–1^, highlighting their “acidic” character and also corroborating their accessibility. However, it is important to note that no remarkable differences were found between the samples concerning the type of acidity- and temperature-dependent effects when the CCN stretching vibration modes of the pyridine ring were analyzed (Figure 6). The band at ∼1451 cm^–1^ (ν_19b_) is assigned to pyridine interacting with Lewis acid sites (LAS). When the temperature is increased to 250 °C, the intensity of this band decreases and shifts towards higher wavenumbers (∼1455 cm^–1^) for both samples, pointing at the existence of acid sites with different strengths. For a deeper understanding of the phenomenon, the region at 1635–1595 cm^–1^ (ν_8a_) was also evaluated, where two clear bands are observed at 150 °C. The former (∼1624 cm^–1^) is associated with pyridine bonded to the coordinatively unsaturated Al^3+^ ions in a tetrahedral environment, which are the strongest LAS in both samples.^7,58^ The latter (∼1616 cm^–1^) could be instead attributed to the pyridine species interacting with either Al^IV^–Al^VI^ bridging vacancies or Al^IV^ located close to a cation vacancy and is ascribed to medium-strength LAS.^55,59^ Interestingly, the sample calcined at 550 °C seems to have a higher amount of strong LAS than the one calcined at 350 °C. This notion is corroborated by the temperature-programmed desorption experiments of ammonia (NH_3_-TPD; Figure S13). Finally, the overall concentration of acid sites was determined via NH_3_-TPD, resulting in 35 and 48 μmol g^–1^ for the sample calcined at 350 and 550 °C, respectively. Ultimately, no indication of Brønsted acid sites (band of adsorbed pyridine expected around 1545 cm^–1^) was found for any of the samples. The acidity of the samples will be further investigated when exploring potential catalysis applications of the materials.

## 3 Conclusions

Mechanochemical synthesis gives easy access to otherwise hard-to-synthesize materials. Highly pure tohdite nanopowders were mechanochemically synthesized by carefully optimizing reaction conditions involved in the dehydration of boehmite. Ball milling at 400 rpm for 48 h led to about 80% selectivity for tohdite, according to the thermogravimetric analysis of the samples. Using AlF_3_ as an additive, the selectivity for tohdite can be increased resulting in an almost phase-pure material. These conditions are relatively mild, in contrast with the extremely harsh ones usually required for the hydrothermal synthesis of tohdite.

Several bulk characterization methods indicate that tohdite is the primary crystalline phase of the resulting material, with an amorphous fraction and corundum present only in small amounts. Furthermore, the tohdite phase is represented by about spherical nanoparticles of average size around 13 nm, randomly packed in large aggregates, as emerged from the direct observation at the electron microscope. The small particle size accounts for the high surface area (∼102 m^2^ g^–1^) detected via N_2_-sorption experiments.

The use of AlF_3_ suppresses the formation of the most stable α-Al_2_O_3_ upon ball milling, further enhancing the phase purity of the material. The role of AlF_3_ is still not well understood, but it appears that some similarities exist between the mechanochemical and hydrothermal syntheses, including the relative stability of phases, i.e., γ-AlOOH <γ-Al_2_O_3_ <5Al_2_O_3_·H_2_O <α-Al_2_O_3_. Progress in this sense is highly appealing because the mechanochemical synthesis of intermediates and metastable phases indicates that more direct methodologies are available to produce hard-to-synthesize materials.

The transformation sequence 5Al_2_O_3_·H_2_O → κ-Al_2_O_3_ → α-Al_2_O_3_ has been carefully studied via TG-DSC. The results indicate that both transformations occur at much lower temperatures than expected, especially the transformation of tohdite into κ-Al_2_O_3_ (445 °C), possibly due to the nanocrystallinity of the material. Furthermore, κ-Al_2_O_3_ was also obtained with a high surface area (88 m^2^ g^–1^ at 550 °C), almost entirely retained at higher temperatures (76 m^2^ g^–1^ at 800 °C). This is an exciting outcome since high values of specific surface area and thus small particle size are unusual for high-temperature metastable alumina phases as it is for the most thermodynamically stable α-Al_2_O_3_. Furthermore, the high hydrothermal stability of tohdite is by itself an important material feature. Therefore, this work could open up new application perspectives for specific, less used aluminum oxides or oxyhydroxides.

Surface and bulk characterization of the samples by FTIR provided further insights into the features of the material and its thermal behavior. Particularly, the results evidenced surface alterations and structural rearrangements upon calcination of the samples, possibly linked to the transformation of tohdite to κ-Al_2_O_3_. Interestingly, tohdite presents hydroxyl groups located both on the surface and in the subsurface layers or bulk of the material. Finally, the presence of Lewis acid sites in both the samples calcined at 350 and 550 °C combined with other attractive material features (e.g., high specific surface area, thermal stability) opens the possibility to investigate their use as catalysts in acid-catalyzed reactions.

## 4 Experimental Section

### 4.1 Materials

Boehmite (γ-AlOOH) was obtained from Sasol (DISPAL 11N7-80, Lot No. S4486J) as a nanopowder (71 m^2^ g^–1^, measured). Aluminum(III) fluoride (AlF_3_) was obtained from Sigma-Aldrich (99.9+%) as an anhydrous powder. All materials were used as received.

### 4.2 Mechanochemical Syntheses

All preparations were carried out using the Fritsch planetary micro mills Pulverisette P7 (classic line). A 45 mL zirconia jar (partially stabilized with 5.5 wt % of Y_2_O_3_) was loaded with 1 g of boehmite. The powder was then milled for varying times (6–96 h of neat milling time) using ten 10 mm Ø zirconia grinding balls (32 g in total) at 400 or 500 rpm. In some cases, the synthesis was carried out in the presence of AlF_3_ (75 mg) as the process control agent (PCA). The milling program implied the repetition of the same two steps cyclically—first, 15 min of milling at the selected frequency and then a 5 min break—until the target milling time was finally reached. Rotation was inverted when moving from one repetition to the other to improve the homogeneity of the treatment. At the end of the milling program, the material was scratched out of the milling jar and thus recovered in nearly quantitative yield. All preparations were carried out at least twice to assess reproducibility.

### 4.3 Thermal Stability Evaluation

The thermal stability of a representative tohdite-rich sample, i.e., specifically the one resulting from 48 h of milling at 400 rpm in the absence of PCA, was investigated by calcination in a static oven. Particularly, about 250 mg of the material was placed in a crucible and heated to 350, 550, 600, 700, 800, 900, or 1000 °C (heating rate, 10 °C min^–1^). The sample was maintained in the furnace for 10 h at the given temperature before allowing the system to cool down to room temperature naturally.

### 4.4 Hydrothermal Stability Evaluation

The hydrothermal stability of the samples was evaluated by dispersing about 0.5 g of the material in 25 mL of deionized water (H_2_O/Al ratio around 150) in a stainless steel autoclave (36 mL). The sealed autoclave containing the suspension of powder in water was heated to 150 °C under stirring and held at this temperature for 24 h after which the system was allowed to cool down naturally to room temperature. The solid was recovered by filtration and then dried in a static oven at 60 °C (overnight).

### 4.5 Characterization Methods

#### 4.5.1 Powder X-ray Diffraction

The X-ray diffraction data were recorded on a Rigaku SmartLab equipped with a rotating anode (9 kW, 45 kV, 200 mA) in the Bragg–Brentano geometry (Cu Kα_1,2_: 1.541 862 Å). An elliptical multilayer mirror was used for generating high-resolution radiation. The samples were placed on a silicon background-free sample holder, and data were collected continuously in the 10–80°2θ range at a scan rate of 0.5° min^–1^ (step size, 0.01°) with a HyPix-3000 multidimensional detector (one-dimensional, 1D mode). The measured patterns were evaluated qualitatively by comparison with entries from the ICDD PDF-2 database and simulated data from the Inorganic Crystal Structure Database (ICSD, FIZ Karlsruhe). The total X-ray scattering analysis data were collected at Petra III (beamline P02.1, DESY, Hamburg, Germany) using a wavelength of 0.207 09 Å. A Varex XRD 4343DT detector was used for data collection. The subsequent pair distribution function (PDF) data were generated with the PDFgetX3 and PDFgui software.^60,61^

#### 4.5.2 N_2_ Physisorption

Nitrogen adsorption–desorption measurements were performed on a Quantachrome NOVA 3200e surface area analyzer at −196 °C. Before the measurements, all of the samples were degassed under vacuum for 1 h at 350 °C. Brunauer–Emmett–Teller (BET) surface areas were calculated in the 0.05–0.20 relative pressure range. The pore size distribution (PSD) was derived from the experimental isotherm using the Barrett–Joyner–Halenda (BJH) method (N_2_-desorption).

#### 4.5.3 Electron Microscopy

High-resolution transmission electron micrographs were collected with a Thermo Scientific Talos F200X (S)TEM microscope equipped with a SuperX EDS system at an acceleration voltage of 200 kV. The samples were prepared by dropping the suspension obtained after 20 min of sonication in ethanol on a lacey carbon-coated copper TEM grid and then dried under a halogen lamp. High-angle annular dark-field scanning-transmission electron (HAADF-STEM) micrographs and energy-dispersive X-ray spectroscopy (EDX) elemental maps were acquired on a Cs probe-corrected Hitachi HD-2700 microscope equipped with a cold field emission gun and two EDAX Octane T Ultra W EDX detectors at an acceleration voltage of 200 kV. Conventional high-resolution transmission electron (HR-TEM) micrographs were collected with this same machine. Finally, the scanning mode imaging was also performed. The samples were usually prepared by sprinkling dry specimens on the TEM grid. Additional studies were carried out with a Hitachi HF-2000 transmission electron microscope equipped with a cold field emission gun and a NORAN energy-dispersive X-ray (EDX) unit at an acceleration voltage of 200 kV. In all cases, the particle size distribution was determined by estimating the diameters of at least 600 particles from several images of the same sample. Elemental composition was determined via energy-dispersive X-ray (EDX) bulk analysis performed on a Hitachi TM3030 PLUS table-top scanning electron microscope (SEM) equipped with an Xplore Compact 30 detector from Oxford Instruments and operated at an acceleration voltage of 15 kV. All of the samples for SEM measurements were prepared by sprinkling dry specimens on a C-film.

#### 4.5.4 Thermogravimetric Analysis

Simultaneous thermal analysis (TG-DSC) coupled with mass spectrometry (MS) was carried out with a Netzsch STA 449 F3 Jupiter thermal analysis instrument connected to a Netzsch QMS 403 D Aeolos mass spectrometer. Approximately 10 mg of sample was heated from 40 to 1200 °C (heating rate, 10 °C min^–1^) under a continuous flow of synthetic air (gas flow rate, 40 mL min^–1^) and an additional protective flow of argon (gas flow rate, 20 mL min^–1^). Mass spectra were collected in the multiple ion detection (MID) mode.

#### 4.5.5 Solid-State NMR

The solid-state NMR spectra were recorded on a Bruker AVANCE III-HD 500WB spectrometer operating at a resonance frequency of 130.3 MHz (for ^27^Al) or 500.2 MHz (for ^1^H) using a double bearing magic angle spinning (MAS) probe (DVT BL4) configured for 4.0 mm (o.d.) rotors. For ^19^F MAS NMR, the high-frequency channel of a 4 mm-CP-MAS DVT double-resonance probe head (BL4 Ag-C/H-F) tuned at 470.65 MHz was used. The ^27^Al MAS NMR spectra were measured by applying single excitation pulses (0.6 μs) with a recycle delay of 1 s (64 scans) at a spinning rate of 13 kHz. The ^19^F MAS NMR spectra were measured by applying single excitation pulses (3 μs) with a recycle delay of 5 s (64 scans) at a spinning rate of 4 kHz. The ^1^H MAS NMR spectra were measured by applying single excitation pulses (3 μs) with a relaxation delay of 5 s (4000 scans) at a spinning rate of 13 kHz. The chemical shifts are reported relative to tetramethylsilane (TMS) for both ^1^H and ^27^Al (indirect referencing). Before the measurements, the samples were subjected to an activation procedure (200 °C overnight in a drying oven or 350 °C for 1 h under a high vacuum, as specified in the Supporting Information) to remove physisorbed water molecules detrimental to the ^1^H NMR analysis. After activation, the samples were transferred into the rotor under an Ar atmosphere. Thus, about 150 mg of material was transferred and closely packed into zirconia (yttria-stabilized) rotors sealed with a Kel-F turbine cap (or a Vespel-type cap in the case of F-containing samples). The integration analysis was performed using the line-fitting tool “sola”, i.e., a module of Topspin 3.6. After baseline correction using the cubic spline method, the shape of up to three Gaussian lines was optimized to fit the spectra within the range of chemical shift expected for magnetically inequivalent Al species. Then, the cumulated area of the fit Gaussian lines was used to estimate the relative fraction of the said magnetically inequivalent Al species.

#### 4.5.6 NH_3_-Temperature-Programmed Desorption

Temperature-programmed desorption of ammonia (NH_3_-TPD) was performed on a Micromeritics Autochem II 2920 device. First, 80–90 mg of sample was activated at 350 or 550 °C for 1 h (heating rate, 10 °C min^–1^) in Ar (flow rate, 50 mL min^–1^) and then cooled to 150 °C. The activation temperature matches the temperature at which the samples were previously calcined. Next, the sample was exposed to NH_3_ (10% NH_3_ in He at 75 mL min^–1^) for 30 min (saturation) and subsequently purged with He (flow rate, 75 mL min^–1^) for 1 h to remove the physisorbed species. The NH_3_-desorption profiles were measured in the temperature range from 100 °C to 350 or 550 °C (heating rate, 10 °C min^–1^) in He (flow rate, 50 mL min^–1^). Again, the maximum temperature matches the temperature at which the samples were initially calcined. The baseline correction was performed using blank experiments. The quantitative analysis of data was carried out with integrated software tools.

#### 4.5.7 Infrared Spectroscopy

The evolution of hydroxyl groups with the temperature was studied in a Harrick Praying Mantis diffuse reflection (DRIFT) accessory equipped with a reaction chamber (CaF_2_ windows), where the powder sample was placed. First, the chamber was flushed with nitrogen for 60 min to remove the moisture. After that, the sample was heated from room temperature to 500 °C (rate, 10 °C min^–1^) and then kept at this temperature for 10 h. Spectra were collected stepwise every 50 °C, until 500 °C, and every hour afterward using a Nicolet Magna-IR 560 spectrometer with a mercury–cadmium–telluride (MCT) detector. Measurements were carried out in the 4000–1000 cm^–1^ range with a 4 cm^–1^ resolution (100 scans per spectrum). Attenuated total reflectance-Fourier transform infrared (ATR-FTIR) spectroscopy (diamond crystal) was used to investigate the skeletal features of the samples through a Nicolet Magna-IR 560 spectrometer with an MCT detector (4 cm^–1^ resolution, 64 scans per spectrum). Isotopic H/D exchange and characterization of acid sites were assessed in a homemade quartz cell (transmission sampling technique, CaF_2_ windows). Particularly, the evolution of probe molecules (D_2_O or pyridine) was monitored with a Nicolet iS50 FTIR spectrometer equipped with an MCT detector. Spectra were recorded in the 4000–1200 cm^–1^ range (4 cm^–1^ resolution, 128 scans per spectrum) at room temperature and normalized to a pellet density of 10 mg cm^–2^. Samples were pressed into pellets without adding any additional matrix (e.g., KBr) and outgassed in situ at 350 °C for 8 h under vacuum. Deuteration of the samples was carried out by introducing 15–20 mbar of D_2_O vapor (30 min) into the system at room temperature, followed by outgassing at 350 °C (30 min). This procedure was repeated four times until no significant changes were observed between two consecutive measurements. Type and strength of acid sites were evaluated after adsorbing 3 mbar of pyridine vapor at 150 °C (20 min) and then outgassing at 150, 250, and 350 °C (20 min at each temperature). Spectra of adsorbed pyridine were obtained by subtracting the spectrum before pyridine adsorption from the one after pyridine adsorption and baseline-corrected.

## Supporting Information Available

Powder XRD and PDF of tohdite samples (Figure S1); ^1^H and ^27^Al MAS NMR spectra of tohdite samples before and after calcination at 350 and 550 °C (Figure S2); summary of ^1^H and ^27^Al solid-state NMR characterization of the samples (Table S1); STEM and TEM micrographs of tohdite (Figure S3); TG curves of tohdite samples before and after activation at 350 °C (Figure S4); TEM micrograph, EDX line scans, and ^19^F and ^27^Al MAS NMR spectra of tohdite samples obtained in the presence of AlF_3_ (Figure S5); N_2_-sorption isotherm, nanoparticle size distribution (histogram), TG/DSC curve, and ^1^H MAS NMR of tohdite samples obtained in the presence of AlF_3_ (Figure S6); powder XRD patterns of tohdite samples after calcination at 350, 550, 600, 700, 800, 900, and 1000 °C (Figure S7); ^1^H and ^27^Al MAS NMR of tohdite samples after calcination at 800 °C (Figure S8); specific surface area of the samples *vs*. calcination temperature (Figure S9); in situ DRIFT spectra of the samples calcined at 350, 550, and 800 °C measured at different temperatures (Figure S10); skeletal FTIR spectra (ATR) of the samples calcined at 350 and 550 °C (Figure S11); transmission FTIR spectra of the samples calcined at 350 and 550 °C before and after exposure to pyridine (Figure S12); NH_3_-TPD of the samples calcined at 350 and 550 °C (Figure S13); and powder XRD patterns of tohdite samples before and after the hydrothermal treatment (Figure S14) (PDF)
