# Supplementary material for: Surface and Bulk Chemistry of Mechanochemically Synthesized Tohdite Nanoparticles
Source: J Am Chem Soc. 2022 May 23;144(21):9421–33. doi: 10.1021/jacs.2c02181 (PMC9164225; doi:10.1021/jacs.2c02181)
Supplement: Supplementary file 1 — ja2c02181_si_001.pdf [file ja2c02181_si_001.pdf]

Supporting Information

to

Surface and Bulk Chemistry of Mechanochemically Synthesized Tohdite Nanoparticles

Jacopo De Bellis, Cristina Ochoa-Hernández, Christophe Farès, Hilke Petersen, Jan Ternieden, Claudia Weidenthaler, Amol P. Amrute, and Ferdi Schüth*

Max-Planck-Institut für Kohlenforschung,
Kaiser-Wilhelm-Platz 1, D-45470 Mülheim an der Ruhr, Germany.

* Corresponding Author (e-mail: schueth@kofo.mpg.de)

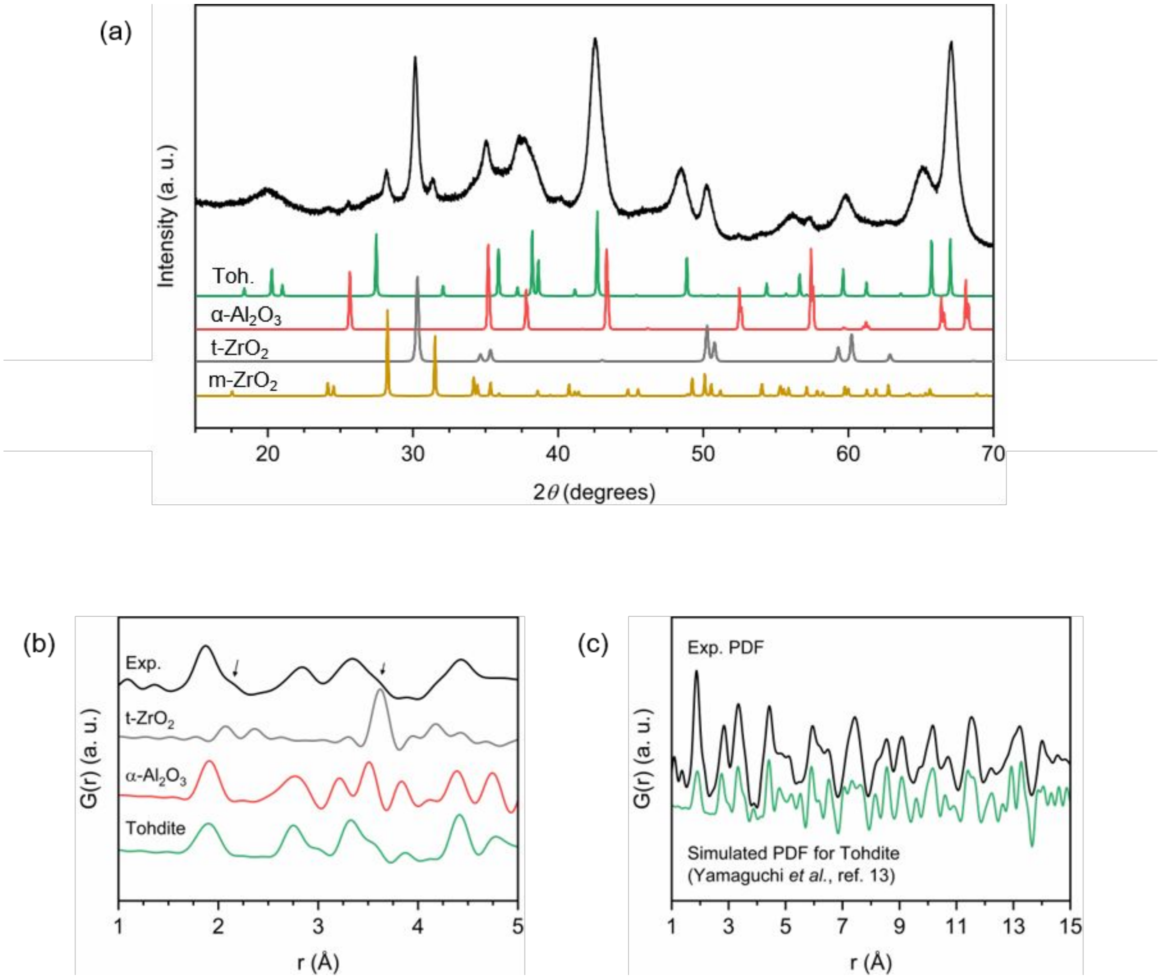

Figure S1. (a) XRD pattern (measured, black line) of the material resulting from the dehydration of boehmite (γ-AlOOH) upon 48 h of ball milling at 400 rpm as compared to the simulated patterns for $5Al_2O_3\%H_2O$ (tohdite), α-Al_2O_3 (corundum), *t*-ZrO_2, and *m*-ZrO_2. (b) Comparison of the experimental PDF for the same sample and the simulated PDFs for $5Al_2O_3\%H_2O$ (tohdite), α-Al_2O_3 (corundum), and *t*-ZrO_2 in the short range of distances (from 1 to 5 Å). (c) Comparison of the experimental PDF for the tohdite sample and the simulated PDFs for $5Al_2O_3\%H_2O$ (tohdite) in the 1 to 15 Å range of distances. The structural model from Yamaguchi *et al.* (ref. 13, manuscript) was used to simulate the XRD pattern and PDF of $5Al_2O_3\%H_2O$ (tohdite).

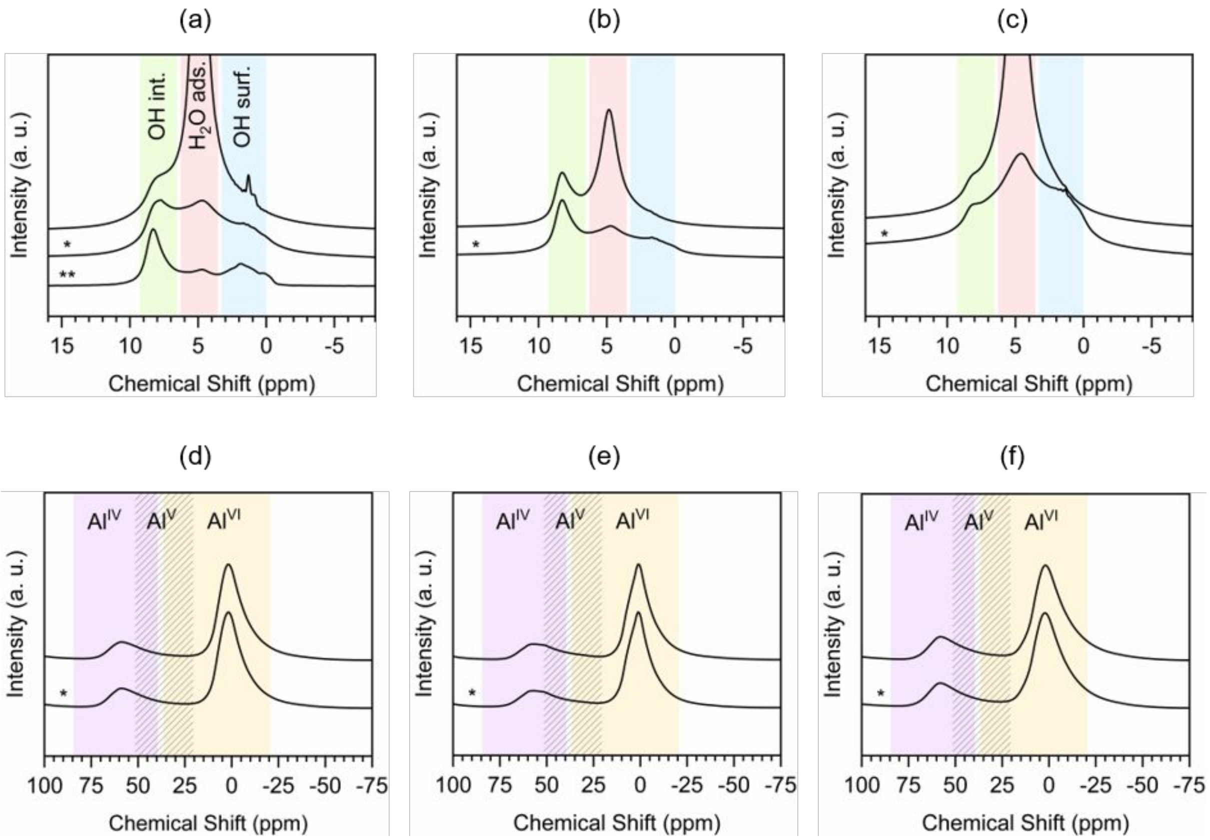

Figure S2. ^{1}H (a-c) and ^{27}Al MAS NMR (d-f) spectra of as-synthesized tohdite (a, d), after calcination at 350 °C for 10 h (b, e), and 550 °C for 10 h (c, f). The ^{1}H and ^{27}Al MAS NMR measurements were repeated after activation at 200 °C overnight (*) or at 350 °C for 1 h under a high vacuum (**). Whereas the untreated samples were exposed to air, all activated samples were manipulated under Ar atmosphere. ^{1}H NMR peak assignments follow the color code specified in detail (a).

Table S1. Summary of ^1H and ^{27}Al MAS NMR characterization of the materials resulting from the dehydration of boehmite (γ-AlOOH) upon ball milling under varying conditions and subsequent calcination as specified under *Sample Description*. In detail, ^1H and ^{27}Al NMR chemical shifts (δ) and corresponding assignments are reported. To improve the ^1H MAS NMR activation of the samples was carried out, i.e., at 200 °C (overnight in a drying oven, *) or 350 °C (1 h under a high vacuum), resulting in different chemical shifts, i.e., δ* and δ**, respectively. Activation did not affect the ^{27}Al MAS NMR chemical shifts. The relative fraction and ratio of Al(IV) and Al(VI) species are reported.

**Sample Description**	**^1H MAS NMR**		**^{27}Al MAS NMR**		
	Peak Position and Assignment		*Peak Position and Assignment*		*Al(IV) / Al(VI)*
	OH bulk	*H_2O ads.*	*Al(IV)*	*Al(VI)*	
Tohdite + AlF_3<br>*400 rpm, 48 h, with AlF_3*	δ, 7.8 ppm<br>δ*, 7.4 ppm<br>δ**, 7.9 ppm	δ, 5.1 ppm<br>δ*, 4.8 ppm<br>δ**, 4.8 ppm	δ, 57.8 ppm<br>(18%)	δ, 1.8 ppm<br>(82%)	0.22
Tohdite + α-Al_2O_3 (trace)<br>*400 rpm, 48 h, without AlF_3*	δ, 7.9 ppm<br>δ*, 7.9 ppm<br>δ**, 8.3 ppm	δ, 4.9 ppm<br>δ*, 4.9 ppm<br>δ**, 4.7 ppm	δ, 58.7 ppm<br>(19%)	δ, 1.6 ppm<br>(81%)	0.24
Tohdite + α-Al_2O_3 (trace)<br>*400 rpm, 48 h, without AlF_3*<br>*calc. 350 °C, 10 h*	δ, 8.2 ppm<br>δ*, 8.2 ppm	δ, 4.8 ppm<br>δ*, 4.9 ppm	δ, 56.6 ppm<br>(21%)	δ, 0.9 ppm<br>(79%)	0.27
κ-Al_2O_3 + α-Al_2O_3 (trace)<br>*400 rpm, 48 h, without AlF_3*<br>*calc. 550 °C, 10 h*	δ, 8.0 ppm<br>δ*, 8.3 ppm	δ, 4.9 ppm<br>δ*, 4.7 ppm	δ, 57.8 ppm<br>(18%)	δ, 1.8 ppm<br>(82%)	0.22
κ-Al_2O_3 + α-Al_2O_3 (trace)<br>*400 rpm, 48 h, without AlF_3*<br>*calc. 800 °C, 10 h*	δ, 7.7 ppm<br>δ*, 7.7 ppm	δ, 4.9 ppm<br>δ*, 5.0 ppm	δ, 57.8 ppm<br>(17%)	δ, 1.8 ppm<br>(83%)	0.21

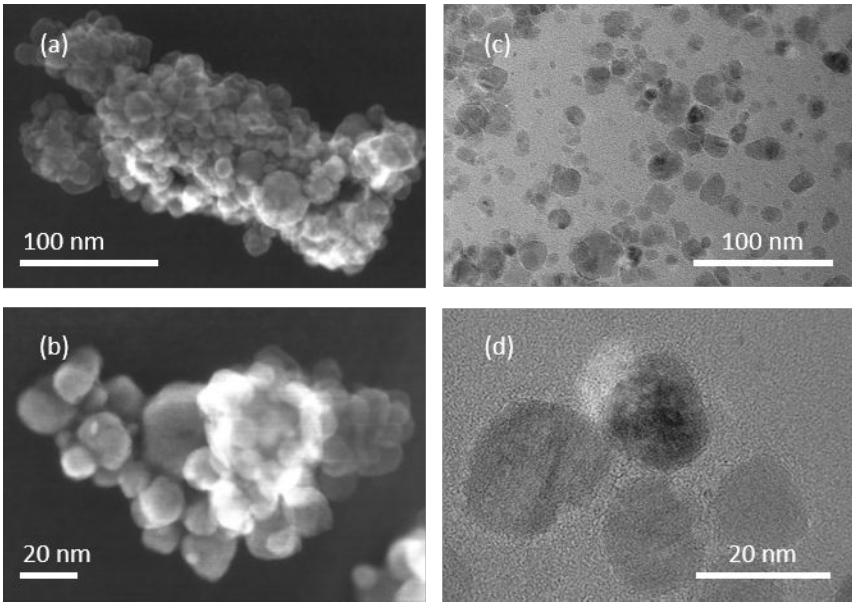

Figure S3. STEM (a-b, scanning mode) and conventional TEM (c-d) micrographs of tohdite nanoparticles.

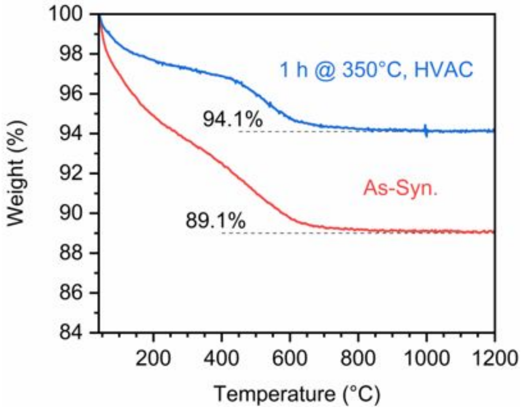

Figure S4. Thermogravimetric curves of tohdite samples measured before (as-synthesized material) and after activation for 1 h at 350°C under high vacuum (HVAC). The residual weight at 1200 °C is reported for each sample.

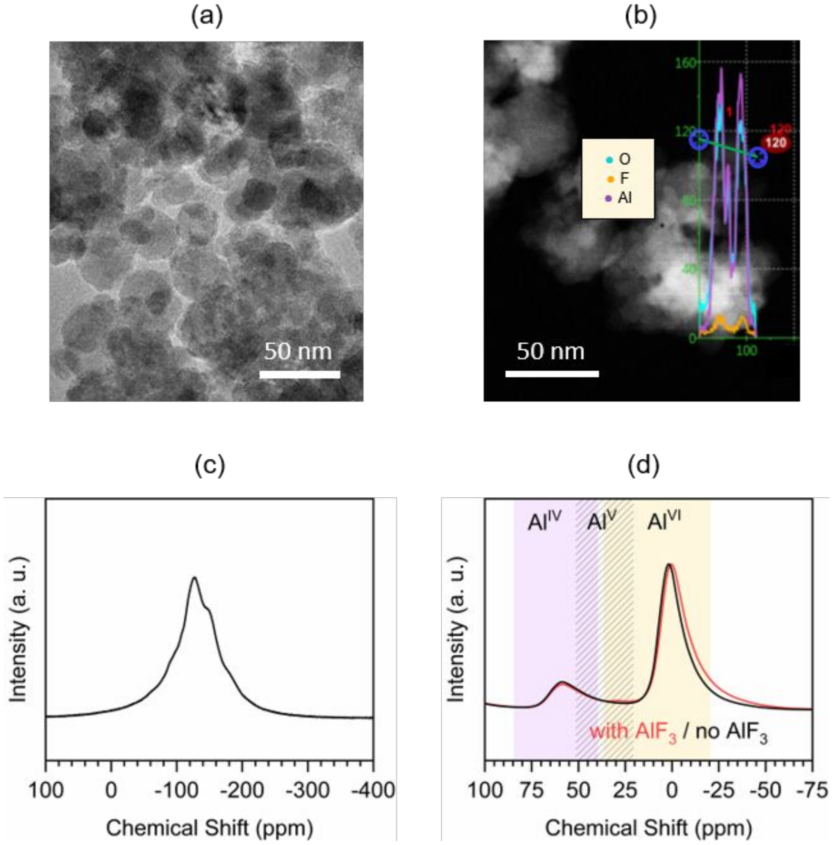

Figure S5. (a) Representative HR-TEM micrograph of the material resulting from the dehydration of boehmite (γ-AlOOH) upon 48 h of milling at 400 rpm in the presence of AlF_3 as the process control agent. (b) Selected HAADF-STEM micrograph and superimposed EDX line scan intensity plot, i.e., signal intensity for O, Al, and F *vs.* relative position along the line where the signal was measured. (c) ^{19}F MAS NMR spectrum of the same sample. (d) ^{27}Al MAS NMR spectra of the materials resulting from the dehydration of boehmite (γ-AlOOH) upon 48 h of milling at 400 rpm, with (red line) and without (black line) AlF_3 as the process control agent, respectively. Typical ranges for tetra-, penta-, and hexacoordinated Al species are highlighted.

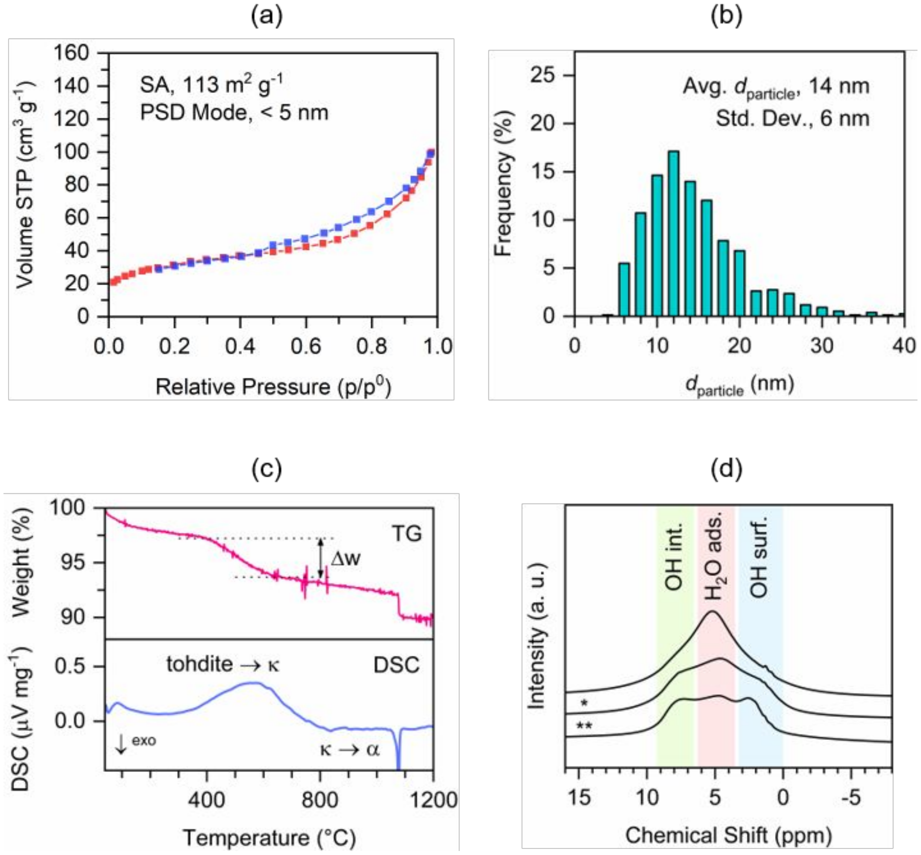

Figure S6. Characterization data for the material resulting from the dehydration of boehmite (γ-AlOOH) upon 48 h of milling at 400 rpm in the presence of AlF_3 as the process control agent. (a) N_2-sorption isotherm. Specific surface area (BET method) and mode of the pore size distribution (PSD, BJH method) are reported. (b) Particle size distribution (histogram). Average particle size (diameter) and standard deviation are attached. (c) TG and DSC curves measured after activation at 350 °C for 1 h under a high vacuum. The weight loss (Δw ≈ −3.3%) observed from the onset (i.e., 415 °C) of the transformation of tohdite to κ-Al_2O_3 is highlighted. (d) From top to bottom, ^1H MAS NMR spectra of the as-synthesized material, measured before (no mark) and after activation at 200 °C overnight (*) or at 350 °C for 1 h under a high vacuum (**). Peak assignments are specified.

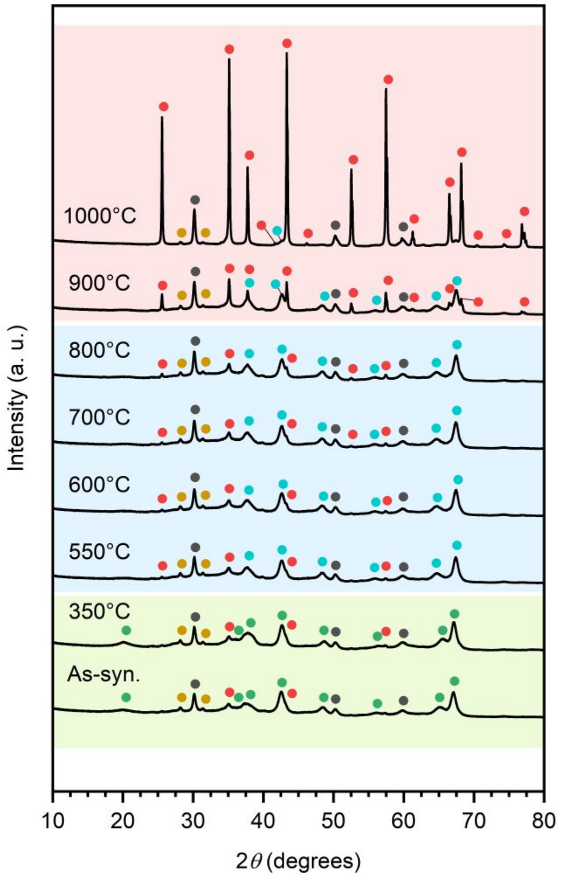

Figure S7. XRD patterns of the materials resulting from the calcination (ex-situ) of tohdite nanoparticles for 10 h at the given temperatures. The most prominent reflections of the crystalline phases present in the sample are marked with symbols as follows: • α-Al_2O_3 (corundum), • κ-Al_2O_3, • $5Al_2O_3$%H_2O (tohdite), • *t*-ZrO_2 (stabilized), • *m*-ZrO_2.

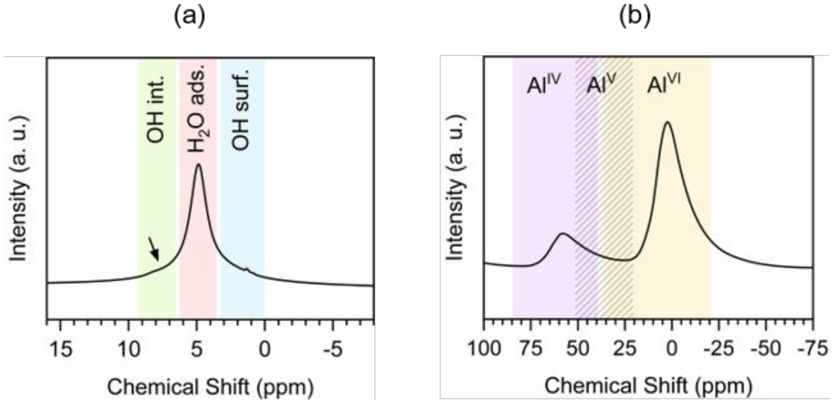

Figure S8. ^{1}H (a) and ^{27}Al MAS NMR (b) spectra of the samples calcined at 800 °C for 10 h.

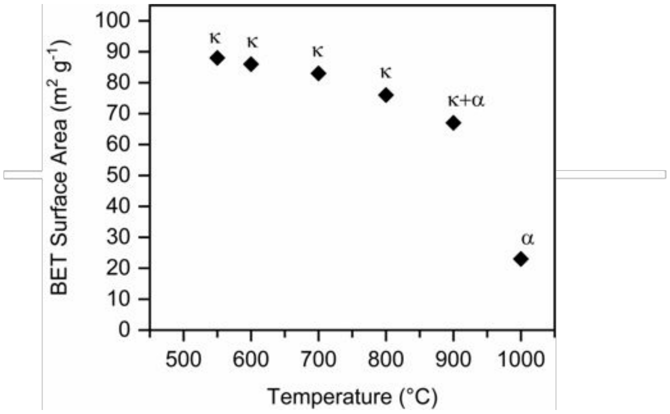

Figure S9. Brunauer-Emmett-Teller (BET) surface area of calcined tohdite samples is plotted against corresponding calcination temperatures. In each case, calcination was carried out for 10 h at the given temperature (more detailed information is provided in the *Experimental Section*). The predominant alumina phase(s) (from XRD, Fig. S6a) is specified for each temperature, typically κ-Al_2O_3 and/or α-Al_2O_3.

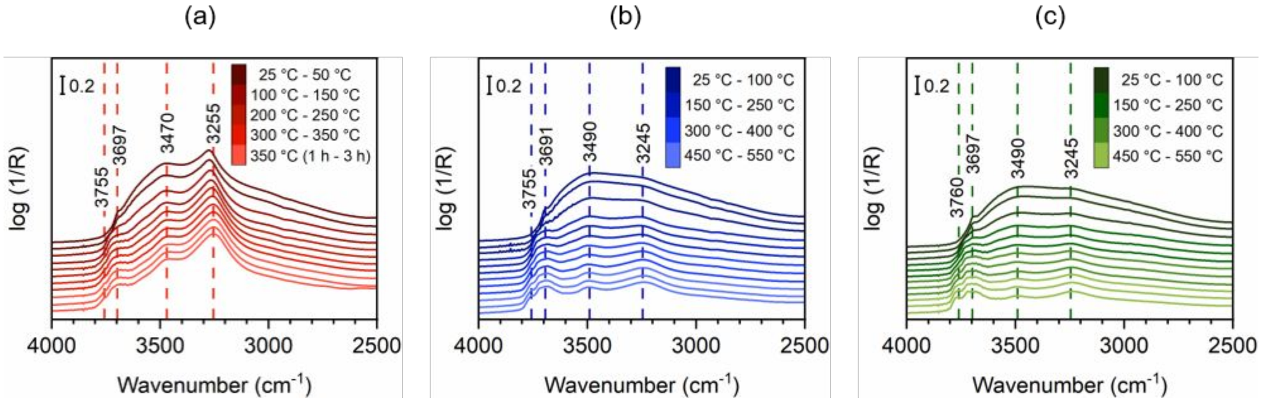

Figure S10. *In situ* DRIFT spectra in the range typical for hydroxyl groups stretching of the samples calcined at 350 °C (a), 550 °C (b), and 800 °C (c) for 10 h, measured at different temperatures. The color guide is attached to the plot.

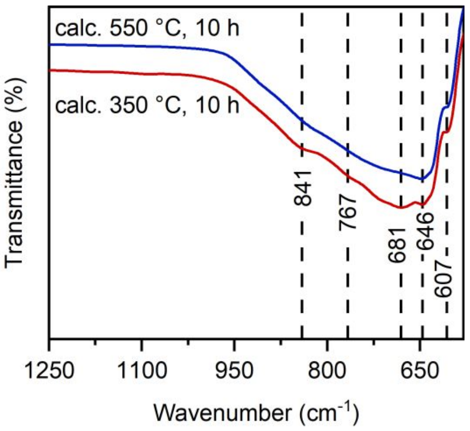

Figure. S11. Skeletal FTIR spectra (ATR) of the samples calcined at 350 °C (red) and 550 °C (blue) for 10 h.

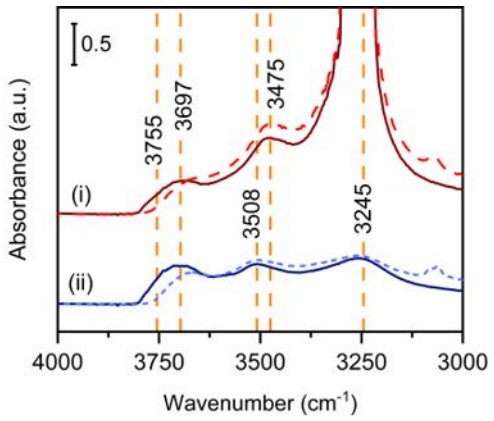

Figure S12. Transmission FTIR spectra in the range typical for hydroxyl groups stretching of the samples calcined at 350 °C (i) and 550 °C (ii) for 10 h. Solid and dashed lines correspond to the spectra measured after outgassing the samples at 350 °C for 8 h and outgassing followed by exposure to 3 mbar of pyridine, respectively.

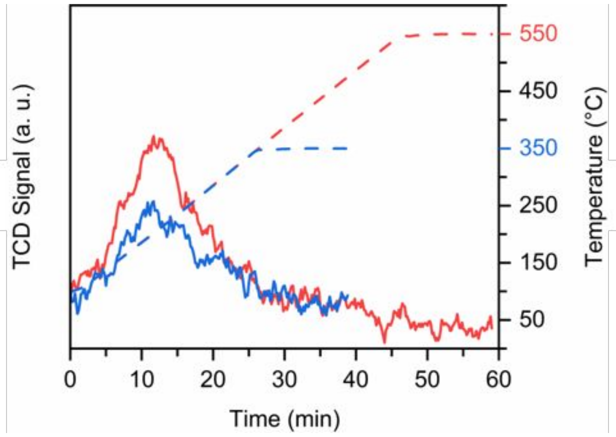

Figure S13. Temperature-programmed desorption of ammonia (NH_3-TPD) over the samples calcined at 350 °C (blue) and 550 °C (red) for 10 h. The TCD signal (solid lines) and temperature trend (dotted lines) are plotted against measuring time, respectively.

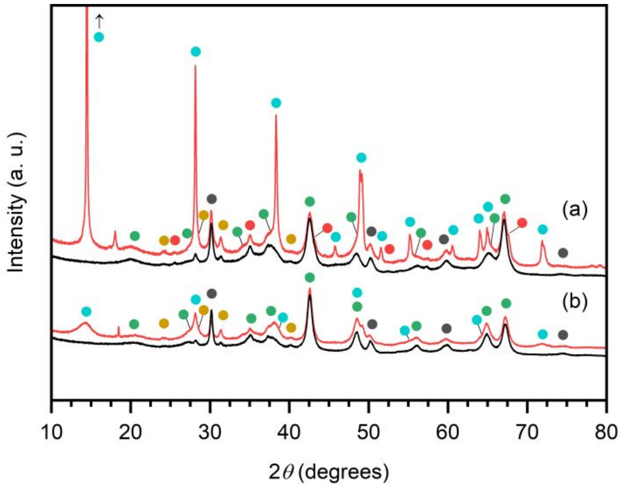

Figure S14. XRD patterns of the material resulting from 48 h of milling at 400 rpm without AlF_3 (a) and 24 h of milling at 500 rpm with AlF_3 as the process control agent before (black lines) and after (red lines) the hydrothermal treatment, i.e., 24 h in deionized water at 150 °C (H_2O / Al ratio of about 150, more information in *Experimental Section*).
